# Supplementary material for: Infection and telomere length: A systematic review
Source: PLoS One. 2025 Sep 23;20(9):e0333107. doi: 10.1371/journal.pone.0333107 (PMC12456831; doi:10.1371/journal.pone.0333107)
Supplement: S3 File — (DOCX) [file pone.0333107.s003.docx]

**Searches**

All databases were searched twice:

Initial search- conception to August 31, 2023

Updated search- August 31, 2023 to May 22, 2025

**Database: Ovid MEDLINE(R) ALL**
**Search Strategy:**
**1**  (infect* or pathogen or virus* or viral or bacteri* or parasit* or communicable disease*).mp.
**2**  exp Infections/
**3**  (telomer* or TTAGGG* or chromosome end* or chromosome cap* or end-replication problem or end-replication malfunction* or end-replication issue* or end-replication impairment* or end-replication failure*).ti,ab.
**4**  Telomere Shortening/
**5**  Telomere/
**6**  ((case* adj5 control*) or (case adj3 comparison*) or control group* or cohort or longitudinal or prospective or retrospective).ti,ab. or "clinical trial".pt. or "clinical trial, phase i".pt. or "clinical trial, phase ii".pt. or clinical trial, phase iii.pt. or clinical trial, phase iv.pt. or controlled clinical trial.pt. or "multicenter study".pt. or "randomi?ed controlled trial".pt. or ((randomi?ed adj7 trial*) or (controlled adj3 trial*) or (clinical adj2 trial*) or ((single or doubl* or tripl* or treb*) and (blind* or mask*))).ti,ab,kw. or ("4 arm" or "four arm").ti,ab,kw. or (cross-sectional or prevalence or transversal).ti,ab,kw. or mendelian randomi?ation.ti,ab. or control patients.mp. or control subjects.mp. or control participants.mp. or patient*.ti,ab. or subjects.ti,ab. or Case-Control Studies/ or Control Groups/ or Matched-Pair Analysis/ or Cohort Studies/ or Longitudinal Studies/ or Follow-Up Studies/ or Prospective Studies/ or Retrospective Studies/ or Double-Blind Method/ or Clinical Trials as Topic/ or Clinical Trials, Phase I as Topic/ or Clinical Trials, Phase II as Topic/ or Clinical Trials, Phase III as Topic/ or Clinical Trials, Phase IV as Topic/ or Controlled Clinical Trials as Topic/ or Randomized Controlled Trials as Topic/ or "Early Termination of Clinical Trials"/ or Multicenter Studies as Topic/ or Cross-Sectional Studies/ or Prevalence/ or Epidemiologic Studies/ or Mendelian Randomization Analysis/ or Observational Study/
**7**  1 or 2
**8**  3 or 4 or 5
**9**  6 and 7 and 8

**Database: Embase Classic+Embase**
**Search Strategy:**
**1**  (infect* or pathogen or virus* or viral or bacteri* or parasit* or communicable disease*).mp.
**2**  exp Infection/
**3**  (telomer* or TTAGGG* or chromosome end* or chromosome cap* or end-replication problem* or end-replication malfunction* or end-replication issue* or end-replication impairment* or end-replication failure*).ti,ab.
**4**  telomere shortening/
**5**  telomere length/
**6**  telomere/
**7**  ((case* adj5 control*) or (case adj3 comparison*) or control group* or cohort or longitudinal or prospective or retrospective).ti,ab. or "clinical trial".pt. or "clinical trial, phase i".pt. or "clinical trial, phase ii".pt. or clinical trial, phase iii.pt. or clinical trial, phase iv.pt. or controlled clinical trial.pt. or "multicenter study".pt. or "randomi?ed controlled trial".pt. or ((randomi?ed adj7 trial*) or (controlled adj3 trial*) or (clinical adj2 trial*) or ((single or doubl* or tripl* or treb*) and (blind* or mask*))).ti,ab,kw. or ("4 arm" or "four arm").ti,ab,kw. or (cross-sectional or prevalence or transversal).ti,ab,kw. or mendelian randomi?ation.ti,ab. or control patients.mp. or control subjects.mp. or control participants.mp. or patient*.ti,ab. or subjects.ti,ab. or case control study/ or control group/ or cohort analysis/ or longitudinal study/ or follow up/ or prospective study/ or retrospective study/ or double blind procedure/ or "clinical trial (topic)"/ or "phase 1 clinical trial (topic)"/ or "phase 2 clinical trial (topic)"/ or "phase 3 clinical trial (topic)"/ or "phase 4 clinical trial (topic)"/ or "controlled clinical trial (topic)"/ or "randomized controlled trial (topic)"/ or "early termination of clinical trial"/ or "multicenter study (topic)"/ or cross-sectional study/ or prevalence/ or epidemiology/ or Mendelian randomization analysis/
**8**  1 or 2
**9**  3 or 4 or 5 or 6
**10**  7 and 8 and 9

**Database: Global Health**
**Search Strategy:**
**1**  (infect* or pathogen or virus* or viral or bacteri* or parasit* or communicable disease*).mp.
**2**  exp infections/
**3**  exp infection/
**4**  (telomer* or TTAGGG* or chromosome end* or chromosome cap* or end-replication problem* or end-replication malfunction* or end-replication issue* or end-replication impairment* or end-replication failure*).ti,ab.
**5**  telomeres/
**6**  ((case* adj5 control*) or (case adj3 comparison*) or control group* or cohort or longitudinal or prospective or retrospective).ti,ab. or "clinical trial".pt. or "clinical trial, phase i".pt. or "clinical trial, phase ii".pt. or clinical trial, phase iii.pt. or clinical trial, phase iv.pt. or controlled clinical trial.pt. or "multicenter study".pt. or "randomi?ed controlled trial".pt. or ((randomi?ed adj7 trial*) or (controlled adj3 trial*) or (clinical adj2 trial*) or ((single or doubl* or tripl* or treb*) and (blind* or mask*))).ti,ab. or ("4 arm" or "four arm").ti,ab. or (cross-sectional or prevalence or transversal).ti,ab. or mendelian randomi?ation.ti,ab. or control patients.mp. or control subjects.mp. or control participants.mp. or patient*.ti,ab. or subjects.ti,ab. or case-control studies/ or cohort studies/ or longitudinal studies/ or retrospective studies/ or clinical trials/ or randomized controlled trials/ or cross-sectional studies/ or disease prevalence/ or seroprevalence/ or epidemiological surveys/ or observational studies/
**7**  1 or 2 or 3
**8**  4 or 5
**9**  6 and 7 and 8

**Database: Web of Science**
**Search Strategy:**

1: **TS**=(infect* or pathogen or virus* or viral or bacteri* or parasit* or "communicable disease*")

2: TS=(telomer* or TTAGGG* or "chromosome end*" or "chromosome cap*" or "end-replication problem*****" or "end-replication malfunction*****" or "end-replication issue*****" or "end-replication impairment*****" or "end-replication failure*****")

3: TS=((case* **NEAR/**5 control*) or (case **NEAR/3** comparison*) or “control group*” or cohort or longitudinal or prospective or retrospective or (randomi?ed **NEAR/7** trial*) or (controlled **NEAR/3** trial*) or (clinical **NEAR/2** trial*) or ((single or doubl* or tripl* or treb*) and (blind* or mask*)) or "4 arm" or "four arm" or “cross-sectional" or prevalence or transversal or “mendelian randomi?ation” or patient* or subjects)

4: **TS**= (“control patients” or “control subjects” or “control participants”)

5: #4 OR #3

6: #5 AND #2 AND #1

**Database: SCOPUS**
**Search Strategy:**

Each search separated by ‘AND’

| **Search within** | **Search** |
| --- | --- |
| **Article title, Abstract, Keywords** | infect* OR pathogen OR virus* OR viral OR bacteri* OR parasit* OR {communicable disease*} |
| Article title, Abstract, Keywords | telomer* OR ttaggg* OR {chromosome end*} OR {chromosome cap*} OR {end-replication problem*} OR {end-replication malfunction*} OR {end-replication issue*} OR {end-replication impairment*} OR {end-replication failure*} |
| Article title, Abstract, Keywords | {case* W/5 control* } OR {case W/3 comparison* } OR {control group*} OR cohort OR longitudinal OR prospective OR retrospective OR {randomi?ed W/7 trial* } OR {controlled W/3 trial* } OR {clinical W/2 trial* } OR ((single OR doubl* OR tripl* OR treb* ) AND ( blind* OR mask* )) OR {4 arm} OR {four arm} OR cross-sectional OR prevalence OR transversal OR {mendelian randomi?ation} OR patient* OR subjects OR participant* |

**Database: Cochrane**
**Search Strategy:**

1. Title Abstract Keyword: infect* or pathogen or virus* or viral or bacteri* or parasit* or **(communicable NEXT disease*)**
2. MeSH descriptor: [Infections] explode all trees
3. Title Abstract Keyword: telomer* or TTAGGG* or (chromosome NEXT end*) or (chromosome NEXT cap*) or (end-replication NEXT problem*) or (end-replication NEXT malfunction*) or (end-replication NEXT issue*) or (end-replication NEXT impairment*) or (end-replication NEXT failure*)
4. MeSH descriptor: [Telomere] explode all trees
5. MeSH descriptor: [Telomere Shortening] explode all trees
6. #1 or #2
7. #3 or #4 or #5
8. #6 and #7
