# Supplementary material for: Infection and telomere length: A systematic review
Source: PLoS One. 2025 Sep 23;20(9):e0333107. doi: 10.1371/journal.pone.0333107 (PMC12456831; doi:10.1371/journal.pone.0333107)
Supplement: S4 File — (DOCX) [file pone.0333107.s004.docx]

**GRADE quality assessment reasons to up- or downgrade**

1. **Risk of bias:**

• Not serious if <50% of studies have an overall ROBINS-E score of high risk of bias or very high risk of bias

• Serious if >50% of studies have an overall ROBINS-E score of high risk of bias or very high risk of bias

• Very serious if >70% of studies have an overall ROBINS-E score of high risk of bias or very high risk of bias

**2. Inconsistency:**

Not serious if have 0, serious if have 1, and very serious if have two of the following:

• Wide variance of point estimates across different studies.

• Minimal overlap of confidence intervals.

**3. Indirectness:**

Not serious if have 0, serious if have 1, and very serious if have two of the following:

• Studies differ in terms of population (e.g. hospitalised patients only vs primary care

patients)

• Studies differ in terms of exposure definition (e.g. for example use different methods to

ascertain infections).

• Studies differ in terms of outcome measures (e.g. cell type, assay used, statistical method etc).

**4. Imprecision:**

Low power and wide confidence intervals

• Serious imprecision: Wide confidence intervals

• Very serious imprecision: Very wide confidence intervals

**5. Publication bias:**

Not serious: Funnel plot shows symmetry; no visual evidence of missing studies or small-study effects.

Serious: Funnel plot shows mild asymmetry or potential missing studies on one side.

Very serious: Funnel plot shows clear asymmetry and pattern consistent with publication bias (e.g. small studies with large effects and missing negative studies).

**6. Upgrading**We considered upgrading the certainty of evidence in two main circumstances:

**Large effect size:**

An upgrade for a strong association was considered when the mean difference in relative telomere length exceeded 0.1 units, assuming direct evidence, minimal confounding, and acceptable risk of bias. A further upgrade for a very strong association was considered when the mean difference exceeded 0.2 units and was supported by precise estimates (i.e. narrow confidence intervals) and low overall study limitations.

**Plausible confounding:**

• We considered upgrading if residual confounding would likely reduce the observed effect. For example, if more unwell individuals were exposed but still had better outcomes, this might suggest the true effect is even greater.

• Conversely, when confounding was likely to exaggerate an effect but no association was seen, this could support the reliability of the null finding.

**7. Overall Certainty**The certainty of evidence was rated as very low, low, moderate, or high, based on the GRADE framework.
