# Supplementary material for: Infection and telomere length: A systematic review
Source: PLoS One. 2025 Sep 23;20(9):e0333107. doi: 10.1371/journal.pone.0333107 (PMC12456831; doi:10.1371/journal.pone.0333107)
Supplement: S1 Table — (DOCX) [file pone.0333107.s005.docx]

**Data extraction form details**

Data extraction was based on the PECOS criteria and the following information was extracted from each study:

| Category | Details extracted |
| --- | --- |
| Population | Age (mean, median or range), sex, setting, recruitment strategy, inclusion and exclusion criteria. |
| Exposure | Chronic or acute, pathogen type, definition and ascertainment, severity, number of exposed. |
| Comparator | Definition and ascertainment, number of comparators. |
| Outcome | Telomere length or attrition, Type of telomere measurement assay used e.g. Quantitative Polymerase Chain Reaction (Q-PCR), Type of telomere measure e.g. relative or absolute length, cell type in which telomere length was measured, number of participants with the outcome. |
| Study Characteristics | Authors, name of study, year of publication, study design, setting, country, duration of follow up. |
| Results extracted | The results extracted included unadjusted mean/ median telomere length measurements as well as the crude and adjusted effect estimates from statistical modelling e.g. beta coefficients from linear regression, odds ratios from logistic regression, F-values from mixed effect models. Data on covariates that were adjusted for was also collected, as well as data on stratified analyses e.g. by sex, age. Severity results were also collected. |
