# Supplementary material for: Infection and telomere length: A systematic review
Source: PLoS One. 2025 Sep 23;20(9):e0333107. doi: 10.1371/journal.pone.0333107 (PMC12456831; doi:10.1371/journal.pone.0333107)
Supplement: S2 Table — (DOCX) [file pone.0333107.s006.docx]

Overall and domain-specific risk of bias scores for each exposure-outcome relationship using the ROBINS-E tool.

| **Author** | **Year published** | **Name of study** | **infection** | **Overall ROB score** | **Direction of bias, Threatens conclusion?** | **Score justification** | **D1-Confounding** | **D2-Exposure measurement** | **D3- Participant selection** | **D4-** **post-exposure interventions** | **D5- Missing data** | **D6-Outcome measurement** | **D7- Result selection** |
| --- | --- | --- | --- | --- | --- | --- | --- | --- | --- | --- | --- | --- | --- |
| Aiello | 2017 | The impact of pathogen burden on leukocyte telomere length in the Multi-Ethnic Study of Atherosclerosis | C. pneumoniae | some | NI, no | lack of information about post exposure interventions and cannot say follow-up begin at (or close to) the start of the exposure window as cross-sectional study. | low | low | some | some | low | low | low |
|  |  |  | HSV-1 | some | NI, no | Lack of information about post exposure interventions and cant say follow-up begin at (or close to) the start of the exposure window as cross-sectional study. | low | low | some | some | low | low | low |
|  |  |  | CMV | some | NI, no | Lack of information about post exposure interventions and cant say follow-up begin at (or close to) the start of the exposure window as cross-sectional study. | low | low | some | some | low | low | low |
|  |  |  | H.Pylori | some | NI, no | Lack of information about post exposure interventions and cant say follow-up begin at (or close to) the start of the exposure window as cross-sectional study. | low | low | some | some | low | low | low |
|  |  |  | Combined burden | some | NI, no | lack of information about post exposure interventions and cannot say follow-up begin at (or close to) the start of the exposure window as cross-sectional study. | low | low | some | some | low | low | low |
| Al-Awadhi | 2024 | Relative Telomere Length in Cervical Exfoliated Cells among Women with High-Risk Human Papillomavirus | HR-HPV | very high | NI, yes | No adjustment for any confounding variables. Unclear selection of controls meaning a high potential for selection bias. Moreover there was a potential for issues with post-exposure interventions. | high | low | high | some | low | low | low |
| Albosale | 2021 | Association of Relative Telomere Length and Risk of High Human Papillomavirus Load in Cervical Epithelial Cells | HR-HPV | very high | NI, yes | There was no evidence of an attempt to adjust for confounding for the infection-telomere length analysis . | high | low | some | some | low | low | low |
| Andreu-Sanchez | 2024 | Antibody signatures against viruses and microbiome reflect past and chronic exposures and associate with aging and inflammation | CMV | very high | NI, yes | It was not clear whether there was any adjustment for confounding variables, moreover the study had issues with potential selection bias and post-exposure interventions. | high | low | some | some | low | low | low |
|  |  |  | Rhinovirus | very high | NI, yes | It was not clear whether there was any adjustment for confounding variables, moreover the study had issues with potential selection bias and post-exposure interventions. | high | low | some | some | low | low | low |
| Auld | 2016 | HIV Infection Is Associated with Shortened Telomere Length in Ugandans with Suspected Tuberculosis | HIV | some | NI, no | Most domains low risk of bias but lack of information about post exposure interventions and cannot determine that follow-up begin at (or close to) the start of the exposure window as cross-sectional study. | low | low | some | some | low | low | low |
|  |  |  | TB | high | NI, yes | Little adjustment for relevant confounders, lack of information about post exposure interventions and cannot determine that follow-up begin at (or close to) the start of the exposure window as cross-sectional study. | high | low | some | some | low | low | low |
| Babu | 2019 | Systemic Inflammation and the Increased Risk of Inflamm-Aging and Age-Associated Diseases in People Living With HIV on Long Term Suppressive Antiretroviral Therapy | HIV | high | NI, yes | Lack of adjustment for important confounders, control and exposed group populations different and issues relating to post-exposure interventions. | high | low | high | some | low | low | low |
| Benetos | 2021 | The Nexus Between Telomere Length and Lymphocyte Count in Seniors Hospitalized With COVID-19 | COVID-19 | very high | NI, yes | There was no evidence of an attempt to adjust for confounding for the infection-telomere length analysis. | high | low | some | some | low | low | low |
| Breen | 2022 | Accelerated aging with HIV begins at the time of initial HIV infection | HIV | some | NI, no | Some concerns relating to inadequate adjustment for confounders. | some | low | low | low | low | low | low |
| Cadinanos | 2024 | Partial Recovery of Telomere Length After Long-term Virologic Suppression in Persons With HIV-1 | HIV | very high | NI, yes | The analysis did not adjust for important confounding variables, moreover the study had issues with potential selection bias, post-exposure interventions | high | low | high | some | low | low | low |
| Chico-Sordo | 2022 | Telomeres and oocyte maturation rate are not reduced by COVID-19 except in severe cases | COVID-19 | very high | NI, yes | There was no evidence of an attempt to adjust for confounding for the infection-telomere length analysis. | high | low | some | high | low | low | low |
| Cobos Jimenez | 2016 | T-Cell Activation Independently Associates With Immune Senescence in HIV-Infected Recipients of Long-term Antiretroviral Treatment | HIV | high | NI, yes | Little attempt to adjust for confounding, high risk of bias relating to potential post exposure interventions and cannot conclude that follow-up began at (or close to) the start of the exposure window. | high | low | some | high | low | low | low |
| Ding | 2018 | Stronger Association between Insomnia Symptoms and Shorter Telomere Length in Old HIV-Infected Patients Compared with Uninfected Individuals | HIV | some | NI, no | Inadequate adjustment for confounders, concerns relating to potential post exposure interventions and we cannot conclude that follow-up began at (or close to) the start of the exposure window. | some | low | some | some | low | low | low |
| Dowd | 2013 | Cytomegalovirus is associated with reduced telomerase activity in the Whitehall II cohort | CMV | some | NI, no | Most domains low risk of bias but study did not adjust for some important confounders, lack of information about post exposure interventions and cannot determine whether follow-up begin at (or close to) the start of the exposure window as cross-sectional study. | some | low | some | some | low | low | low |
| Dowd | 2017 | Persistent Herpesvirus Infections and Telomere Attrition Over 3 Years in the Whitehall II Cohort | CMV | some | NI, no | Lack of information about post exposure interventions and cannot determine whether follow-up begin at (or close to) the start of the exposure window as cross-sectional study. | low | low | some | some | low | low | low |
|  |  |  | HSV-1 | some | NI, no | Lack of information about post exposure interventions and cannot determine whether follow-up begin at (or close to) the start of the exposure window as cross-sectional study. | low | low | some | some | low | low | low |
|  |  |  | HSV-6 | some | NI, no | Lack of information about post exposure interventions and cannot determine whether follow-up begin at (or close to) the start of the exposure window as cross-sectional study. | low | low | some | some | low | low | low |
|  |  |  | EBV | some | NI, no | Lack of information about post exposure interventions and cannot determine whether follow-up begin at (or close to) the start of the exposure window as cross-sectional study. | low | low | some | some | low | low | low |
|  |  |  | Combined burden | some | NI, no | Lack of information about post exposure interventions and cannot determine whether follow-up begin at (or close to) the start of the exposure window as cross-sectional study. | low | low | some | some | low | low | low |
| Freimane | 2021 | Telomere length and mitochondrial DNA copy number in multidrug-resistant tuberculosis | MDR-TB | high | NI, yes | Inadequate adjustment for confounders, concerns relating to potential post exposure interventions and we cannot conclude that follow-up began at (or close to) the start of the exposure window. | high | low | some | some | low | low | low |
| Gaardbo | 2013 | Different immunological phenotypes associated with preserved CD4+ T cell counts in HIV-infected controllers and viremic long term non-progressors | HIV | high | NI, yes | Matching on age,sex, ethnicity but no further adjustment for confounding, high risk of bias relating to potential post exposure interventions and cannot conclude that follow-up began at (or close to) the start of the exposure window. Moreover, authors could have selected 'significant' sub-group results as an overall HIV vs non-HIV analysis was not conducted. | high | some | some | high | low | low | high |
| Giesbrecht | 2014 | Select neurocognitive impairment in HIV-infected women: Associations with HIV viral load, hepatitis C virus, and depression, but not leukocyte telomere length | HIV | high | NI, No | Little attempt to adjust for confounding, high risk of bias relating to potential post exposure interventions and cannot conclude that follow-up began at (or close to) the start of the exposure window. | high | low | some | high | low | low | low |
| Gogia | 2015 | Association of HIV viral load and shorter telomere length | HIV | very high | NI, yes | There was no evidence of an attempt to adjust for confounding for the infection-telomere length analysis. | high | low | some | some | low | low | low |
| Gonzalez-Serna | 2017 | Rapid Decrease in Peripheral Blood Mononucleated Cell Telomere Length After HIV Seroconversion, but Not HCV Seroconversion | HIV | high | NI, yes | Only age-adjusted, high risk of bias relating to potential post exposure interventions and cannot conclude that follow-up began at (or close to) the start of the exposure window. | high | low | some | high | low | low | low |
|  |  |  | HCV | high | NI, yes | Only age-adjusted, high risk of bias relating to potential post exposure interventions and cannot conclude that follow-up began at (or close to) the start of the exposure window. | high | low | some | high | low | low | low |
| Grady | 2013 | HCV-infection is not a major determinant of immunesenescence in aging drug users | HCV | very high | NI, yes | Age matched only, not enough information relating to exposure measurement and missing data, concerns relating to potential post exposure interventions and we cannot conclude that follow-up began at (or close to) the start of the exposure window. | high | high | some | some | high | low | low |
|  |  |  | HIV/HCV co-infection | very high | NI, yes | Age matched only, not enough information relating to exposure measurement and missing data, concerns relating to potential post exposure interventions and we cannot conclude that follow-up began at (or close to) the start of the exposure window. | high | high | some | some | high | low | low |
| Hampras | 2016 | Interaction between cutaneous human papillomavirus infection and telomere length in association with cutaneous squamous cell carcinoma | HPV | very high | NI, yes | There was no evidence of an attempt to adjust for confounding for the infection-telomere length analysis. | high | some | some | some | low | low | low |
| Hartling | 2013 | Impaired thymic output in patients with chronic hepatitis C virus infection | HCV | very high | NI, yes | There was no evidence of an attempt to adjust for confounding for the infection-telomere length analysis. | high | some | high | some | high | low | low |
| Hsieh | 2015 | Shorter telomeres in proliferative CD8+CD28+ T cells may contribute to HIV-mediated immunosenescence | HIV | high | NI, yes | Inadequate adjustment for confounders, concerns relating to potential post exposure interventions, we cannot conclude that follow-up began at (or close to) the start of the exposure window and concerns about potential missing data. | high | low | some | some | high | low | low |
| Huang | 2022 | Association between COVID-19 and telomere length: A bidirectional Mendelian randomization study | COVID-19 | low | NI, no | All domains low risk of bias. | low | low | low | low | low | low | low |
| Huang | 2020 | The relation between Helicobacter pylori immunoglobulin G seropositivity and leukocyte telomere length in US adults from NHANES 1999-2000 | H.Pylori | some | NI, no | Lack of information about post exposure interventions and cannot determine whether follow-up begin at (or close to) the start of the exposure window as cross-sectional study. | low | low | some | some | low | low | low |
| Imam | 2012 | Leukocyte telomere length in HIV-infected pregnant women treated with antiretroviral drugs during pregnancy and their uninfected infants | HIV | some | NI, no | Lack of information about post exposure interventions and cannot determine whether follow-up begin at (or close to) the start of the exposure window as cross-sectional study. | some | low | some | some | low | low | low |
| Jiang | 2022 | Telomere Length and COVID-19 Outcomes: A Two-Sample Bidirectional Mendelian Randomization Study | COVID-19 | low | NI, no | All domains low risk of bias | low | low | low | low | low | low | low |
| Jiang | 2023 | Causal pathway from telomere length to occurrence and 28-day mortality of sepsis: an observational and mendelian randomization study | Sepsis | low | NI, no | All domains low risk of bias. | low | low | low | low | low | low | low |
| Krasnienkov | 2022 | Analysis of Relative Average Length of Telomeres in Leukocytes of Women with COVID-19 | COVID-19 | Very high | NI, yes | Almost no adjustment for confounding (except that the assessment was in women only), moreover concerns over lack of information about post exposure interventions and cannot determine whether follow-up begin at (or close to) the start of the exposure window as cross-sectional study. | high | low | high | some | low | low | low |
| Liu | 2015 | Absolute leukocyte telomere length in HIV-infected and uninfected individuals: evidence of accelerated cell senescence in HIV-associated chronic obstructive pulmonary disease | HIV | some | NI, no | Did not adjust for sex or ethnicity, concerns over lack of information about post exposure interventions and cannot determine whether follow-up begin at (or close to) the start of the exposure window. | some | low | some | some | low | low | low |
| Liang | 2024 | DNA methylation-based telomere length is associated with HIV infection, physical frailty, cancer, and all-cause mortality | HIV | Some | NI, no | Lack of information about post exposure interventions and cannot determine whether follow-up begin at (or close to) the start of the exposure window as cross-sectional study | low | low | some | some | low | low | low |
| Ma | 2016 | Association of telomere length in peripheral leukocytes with chronic hepatitis B and hepatocellular carcinoma | HBV | very high | NI, yes | Little attempt to adjust for confounding, high risk of bias relating to potential post exposure interventions, cannot conclude that follow-up began at (or close to) the start of the exposure window and concerns relating to missing data. | high | some | high | some | high | low | low |
| Macamo | 2024 | Telomere Length in a South African Population Co-Infected with HIV and Helminths | HIV | some | NI, no | Lack of information about post exposure interventions and cannot determine whether follow-up begin at (or close to) the start of the exposure window as cross-sectional study | low | low | some | some | low | low | low |
|  |  |  | Helminths | some | NI, no | Lack of information about post exposure interventions and cannot determine whether follow-up begin at (or close to) the start of the exposure window as cross-sectional study | low | low | some | some | low | low | low |
|  |  |  | HIV + Helminths | some | NI, no | Lack of information about post exposure interventions and cannot determine whether follow-up begin at (or close to) the start of the exposure window as cross-sectional study | low | low | some | some | low | low | low |
| Malan-Muller | 2013 | Shorter Telomere Length - A Potential Susceptibility Factor for HIV-Associated Neurocognitive Impairments in South African Woman | HIV | very high | NI, yes | There was no evidence of an attempt to adjust for confounding for the infection-telomere length analysis. | high | low | some | some | low | low | low |
| Manavalan | 2016 | Abnormal bone acquisition with early-life HIV infection: role of immune activation and senescent osteogenic precursors | HIV | high | NI, yes | Little attempt to adjust for confounding for the results relevant to the present systematic review, there are concerns relating to potential post exposure interventions, also we cannot conclude that follow-up began at (or close to) the start of the exposure window. | high | some | some | some | low | low | low |
| Mehta | 2021 | Telomere length is associated with HIV infection, methamphetamine use, inflammation, and comorbid disease risk | HIV | some | NI, no | Didn't adjust for some potential confounding variables, concerns over lack of information about post exposure interventions and cannot determine whether follow-up begin at (or close to) the start of the exposure window due to cross-sectional nature. | some | low | some | some | low | low | low |
| Meijers | 2013 | Cytomegalovirus contributes partly to uraemia-associated premature immunological ageing of the T cell compartment | CMV | high | NI, yes | Did not adjust for important confounders, concerns over lack of information about post exposure interventions , potential for selection bias and cannot determine whether follow-up begin at (or close to) the start of the exposure window . | high | low | high | some | low | low | low |
| Mongelli | 2021 | Evidence for Biological Age Acceleration and Telomere Shortening in COVID-19 Survivors | COVID-19 | very high | NI, yes | Inadequate adjustment for confounding, issues with selection bias and cannot determine whether follow-up begin at (or close to) the start of the exposure window. | high | low | high | some | low | low | low |
| Muhsen | 2019 | Helicobacter pylori infection, serum pepsinogens as markers of atrophic gastritis, and leukocyte telomere length: a population-based study | H.Pylori | some | NI, no | Lack of information about post exposure interventions and cannot determine whether follow-up begin at (or close to) the start of the exposure window as cross-sectional study. | low | low | some | some | low | low | low |
| Nguyen | 2022 | Biological Aging and Periodontal Disease: Analysis of NHANES (2001-2002) | Periodontitis | very high | NI, yes | There was no evidence of an attempt to adjust for confounding for the infection-telomere length analysis. | high | low | some | some | low | low | low |
| Noppert | 2020 | Pathogen burden and leukocyte telomere length in the United States | HSV-1 | some | NI, no | Lack of information about post exposure interventions and cannot determine whether follow-up begin at (or close to) the start of the exposure window as cross-sectional study. | low | low | some | some | low | low | low |
|  |  |  | HSV-2 | some | NI, no | Lack of information about post exposure interventions and cannot determine whether follow-up begin at (or close to) the start of the exposure window as cross-sectional study. | low | low | some | some | low | low | low |
|  |  |  | CMV | some | NI, no | Lack of information about post exposure interventions and cannot determine whether follow-up begin at (or close to) the start of the exposure window as cross-sectional study. | low | low | some | some | low | low | low |
|  |  |  | H.Pylori | some | NI, no | Lack of information about post exposure interventions and cannot determine whether follow-up begin at (or close to) the start of the exposure window as cross-sectional study. | low | low | some | some | low | low | low |
|  |  |  | HBV | some | NI, no | Lack of information about post exposure interventions and cannot determine whether follow-up begin at (or close to) the start of the exposure window as cross-sectional study. | low | low | some | some | low | low | low |
|  |  |  | combined burden | some | NI, no | Lack of information about post exposure interventions and cannot determine whether follow-up begin at (or close to) the start of the exposure window as cross-sectional study. | low | low | some | some | low | low | low |
| Panczyszyn | *2020* | Telomere length in leukocytes and cervical smears of women with high-risk human papillomavirus (HR HPV) infection | HR-HPV | very high | NI, yes | There was no evidence of an attempt to adjust for confounding for the infection-telomere length analysis. | high | low | some | some | low | low | low |
| Pathai | 2013 | Accelerated biological ageing in HIV-infected individuals in South Africa: a case-control study | HIV | high | NI, yes | Age and sex frequency matched only ( the study states it is adjusted for other variables but it is not clear which effect estimate is adjusted and for which variables), lack of information about post exposure interventions, cannot determine whether follow-up begin at (or close to) the start of the exposure window as cross-sectional study also reporting of results was not done in accordance with established methods i.e. study did not present beta coefficient for linear regression analysis. | high | low | some | some | low | low | some |
| Petrara | 2024 | HIV reservoir and premature aging: risk factors for aging-associated illness in adolescents and young adults with perinatally acquired HIV | HIV | high | NI, yes | The analysis did not adjust for important confounding variables, moreover the study had issues with potential selection bias and post-exposure interventions. | high | low | some | some | low | low | low |
| Retuerto | 2022 | Shorter telomere length is associated with COVID-19 hospitalization and with persistence of radiographic lung abnormalities | COVID-19 | high | NI, yes | Important confounders such as sex and ethnicity were omitted, and people hospitalised for COVID likely to have more co-morbidities than the healthy comparator population. | high | low | high | some | low | low | low |
| Richardson | 2000 | Analysis of telomere length and thymic output in fast and slow/non-progressors with HIV infection | HIV | very high | NI, yes | High potential of bias due to unmeasured confounding, selection bias, differential measurement of the exposure and outcome as well as potential for missing data. | high | some | high | some | low | some | low |
| Saberi | 2019 | Dynamics of leukocyte telomere length in pregnant women living with HIV, and HIV-negative pregnant women: A longitudinal observational study | HIV | high | NI, no | Inadequate adjustment for important confounding variables. | high | low | low | low | low | low | low |
|  |  |  | HCV | high | NI, no | Inadequate adjustment for important confounding variables and potential issues with exposure misclassification. | high | some | low | low | low | low | low |
| Savrun | 2023 | Analysis of telomere length in patients with COVID-19 and investigation into its relationship with clinical- demographic data | COVID-19 | very high | NI, yes | The analysis did not adjust for any confounding variables, moreover the study had issues with potential selection bias and post-exposure interventions. | high | low | some | some | low | low | low |
| Sehl | 2021 | Increased Rate of Epigenetic Aging in Men Living With HIV Prior to Treatment | HIV | low | NI,no | All domains low risk of bias. | low | low | low | low | low | low | low |
|  |  |  | HBV | very high | NI, yes | There was no evidence of an attempt to adjust for confounding for the infection-telomere length analysis. | high | low | low | low | low | low | low |
| Shiau | 2021 | Epigenetic Aging Biomarkers Associated With Cognitive Impairment in Older African American Adults With Human Immunodeficiency Virus (HIV) | HIV | high | NI, no | Little attempt to adjust for confounding and issues with potential misclassification of the exposure. | some | high | some | some | low | low | low |
| Shiau | 2024 | Epigenetic Aging and Musculoskeletal Outcomes in a Cohort of Women Living With HIV | HIV | very high | NI, yes | The analysis did not make any attempt to adjust for confounding variables, moreover the study had issues with potential selection bias and post-exposure interventions | high | low | some | some | low | low | low |
| Soares | 2025 | Effect of SARS-CoV-2 infection on sperm telomere length | COVID-19 | high | NI, yes | The analysis did not adjust for important confounding variables, moreover the study had issues with potential selection bias and post-exposure interventions. | high | low | some | some | low | low | low |
| Song | 2020 | Association of periodontitis with leukocyte telomere length in US adults: a cross-sectional analysis of NHANES 1999 to 2002 | Periodontitis | some |  | Lack of information about post exposure interventions and cannot determine whether follow-up begin at (or close to) the start of the exposure window as cross-sectional study | low | low | some | some | low | low | low |
| Spyridopoulos | 2009 | Accelerated telomere shortening in leukocyte subpopulations of patients with coronary heart disease: role of cytomegalovirus seropositivity | CMV | very high | NI, yes | Age matched only, issues with selection bias as analysis compares people with and without coronary heart disease, cannot determine there was any post exposure interventions and cannot determine whether follow-up begin at (or close to) the start of the exposure window. | high | low | high | some | low | low | low |
| Srinivasa | 2014 | Soluble CD163 is associated with shortened telomere length in HIV-infected patients | HIV | some | NI, no | Some concerns with residual confounding and issues with D3 and D4 as cross-sectional. | some | low | some | some | low | low | low |
| Tachtatzis | 2011 | Hepatitis B virus (HBV) replication is confined to hepatocytes with longer telomeres within livers with accelerated ageing | HBV | high | NI, yes | Only age-matched, issues with selection bias as unclear whether control livers could have liver disease, potential for post-exposure interventions and not enough information to determine whether there was missing data. | high | low | high | some | some | low | low |
| Tahara | 2013 | Telomere length in non-neoplastic gastric mucosa correlates with h. pylori infection, degree of gastritis and non-steroidal anti-inflammatory drugs (NSAIDs) use | H.Pylori | very high | NI, yes | There was no evidence of an attempt to adjust for confounding for the infection-telomere length analysis. | high | low | some | some | low | low | low |
| Toljic | 2023 | HIV-Infected Patients as a Model of Aging | HIV | very high | NI, yes | There was no evidence of an attempt to adjust for confounding for the infection-telomere length analysis. | high | low | some | some | low | low | low |
| Tucker | 2000 | T-cell telomere length maintained in HIV-infected long-term survivors | HIV | very high | NI, yes | Age-matched only, potential for exposure misclassification because controls not tested for HIV, lack of information about post exposure interventions and cannot determine whether follow-up begin at (or close to) the start of the exposure window as cross-sectional study. | high | very high | some | some | low | low | low |
| Usadi | 2016 | Telomere Length, Proviral Load and Neurologic Impairment in HTLV-1 and HTLV-2-Infected Subjects | HTLV-1 | high | NI, yes | Inadequate adjustment for confounders, lack of information about post exposure interventions and cannot determine whether follow-up begin at (or close to) the start of the exposure window. | high | low | some | some | low | low | low |
|  |  |  | HTLV-2 | high | NI, yes | Inadequate adjustment for confounders, lack of information about post exposure interventions and cannot determine whether follow-up begin at (or close to) the start of the exposure window. | high | low | some | some | low | low | low |
| Von Kanel | 2015 | Comparison of telomere length in black and white teachers from South Africa: the sympathetic activity and ambulatory blood pressure in Africans study | HIV | high | NI, yes | Analysis only adjusted for age and sex and did not adjust for other important potential confounding variables such as ethnicity and socioeconomic status, there was some missing data with respect to the outcome and results were based on complete case analysis, lack of information about post exposure interventions and cannot determine whether follow-up begin at (or close to) the start of the exposure window. | high | low | some | some | some | low | low |
| Wang | 2019 | Association between common telomere length genetic variants and telomere length in an African population and impacts of HIV and TB | HIV | high | NI, cannot tell | Some issues relating to adjustment for confounders, potential for misclassification of the exposure, concerns relating to potential post exposure interventions and we cannot conclude that follow-up began at (or close to) the start of the exposure window. | some | high | some | some | low | low | low |
|  |  |  | TB | very high | NI, yes | There was no evidence of an attempt to adjust for confounding for the infection-telomere length analysis | high | low | some | some | low | low | low |
| Wang | 2022 | The association of telomere maintenance and TERT expression with susceptibility to human papillomavirus infection in cervical epithelium | HPV | high | NI, yes | The analysis was not adjusted for important confounders except for age-matching, lack of information about post exposure interventions and cannot determine whether follow-up begin at (or close to) the start of the exposure window, some participants were excluded due to missing outcome data and no sensitivity analysis was conducted to see if there was a difference between those with missing data and non-missing data. | high | low | some | some | some | low | low |
| Wolthers | 1996 | T Cell Telomere Length in HIV-1 Infection: No  Evidence for Increased CD4+ T Cell Turnover | HIV | very high | NI, yes | The analysis did not adjust for important confounding variables, moreover the study had issues with potential selection bias, post-exposure interventions and potential for selection of desirable results. | high | low | high | high | low | low | high |
| Womersley | 2021 | Longitudinal telomere length profile does not reflect HIV and childhood trauma impacts on cognitive function in South African women | HIV | high | NI, yes | Concerns relating to confounding, study recruitment and post-exposure interventions. | high | low | high | high | low | low | low |
| Woods | 2023 | Accelerated epigenetic aging in older adults with HIV disease: associations with serostatus, HIV clinical factors, and health literacy | HIV | very high | NI, yes | There was no evidence of an attempt to adjust for confounding for the infection-telomere length analysis. | high | some | high | some | low | low | low |
| Xu | 2022 | Causal association of epigenetic aging and COVID-19 severity and susceptibility: A bidirectional Mendelian randomization study | COVID-19 | low | NI, no | All domains low risk of bias. | low | low | low | low | low | low | low |
| Yang | 2024 | Chronic and Latent Viral Infections and Leukocyte Telomere Length across the Lifespan of Female and Male Individuals Living with or without HIV | HIV | some | NI, no | Lack of information about post exposure interventions and cannot determine whether follow-up begin at (or close to) the start of the exposure window as cross-sectional study | low | low | some | some | low | low | low |
|  |  |  | CMV | some | NI, no | Lack of information about post exposure interventions and cannot determine whether follow-up begin at (or close to) the start of the exposure window as cross-sectional study | low | low | some | some | low | low | low |
|  |  |  | EBV | some | NI, no | Lack of information about post exposure interventions and cannot determine whether follow-up begin at (or close to) the start of the exposure window as cross-sectional study | low | low | some | some | low | low | low |
|  |  |  | HHV-8 | some | NI, no | Lack of information about post exposure interventions and cannot determine whether follow-up begin at (or close to) the start of the exposure window as cross-sectional study | low | low | some | some | low | low | low |
|  |  |  | HSV-1 | some | NI, no | Lack of information about post exposure interventions and cannot determine whether follow-up begin at (or close to) the start of the exposure window as cross-sectional study | low | low | some | some | low | low | low |
|  |  |  | HSV-2 | some | NI, no | Lack of information about post exposure interventions and cannot determine whether follow-up begin at (or close to) the start of the exposure window as cross-sectional study | low | low | some | some | low | low | low |
|  |  |  | HCV | some | NI, no | Lack of information about post exposure interventions and cannot determine whether follow-up begin at (or close to) the start of the exposure window as cross-sectional study | low | low | some | some | low | low | low |
|  |  |  | Combined burden | some | NI, no | Lack of information about post exposure interventions and cannot determine whether follow-up begin at (or close to) the start of the exposure window as cross-sectional study | low | low | some | some | low | low | low |
| Yoshioka | 2012 | Telomere length in non-neoplastic gastric mucosa and its relationship to H. pylori infection, degree of gastritis and non-steroidal anti-inflammatory drugs (nsaids) Use | H.Pylori | very high | NI, yes | There was no evidence of an attempt to adjust for confounding for the infection-telomere length analysis. | high | low | some | some | low | low | low |
| Zanet | 2014 | Association between short leukocyte telomere length and HIV infection in a cohort study: No evidence of a relationship with antiretroviral therapy | HIV | some | NI, no | Most domains were low risk of bias however the final model was not adjusted for sex as this was ruled out due to lack of association in univariate models, lack of information about post exposure interventions and cannot determine whether follow-up begin at (or close to) the start of the exposure window as cross-sectional study. | some | low | some | some | low | low | low |
|  |  |  | HCV | high | NI, yes | The final model was not adjusted for sex or ethnicity as these were ruled out due to lack of association in univariate models, moreover HCV exposure was not confirmed via testing only diagnosis so there could have been misclassification of the exposure, lack of information about post exposure interventions and cannot determine whether follow-up begin at (or close to) the start of the exposure window as cross-sectional study. | some | high | some | some | low | low | low |
|  |  |  | HBV | very high | NI, yes | There was no evidence of an attempt to adjust for confounding for the infection-telomere length analysis. | high | high | some | some | low | low | low |
| Zhang | 2014 | High-risk human papillomavirus infection associated with telomere elongation in patients with esophageal squamous cell carcinoma with poor prognosis | HR-HPV | very high | NI, yes | There was no evidence of an attempt to adjust for confounding for the infection-telomere length analysis. | high | low | some | some | low | low | low |
| Zhu | 2024 | Causal relationship between telomere length and sepsis: a bidirectional Mendelian randomization study | Sepsis | low | NI, no | All domains low risk of bias. | low | low | low | low | low | low | low |
| Zribi | 2019 | Telomere Length Changes during Critical Illness: A Prospective, Observational Study | Sepsis | very high | NI, yes | There was no evidence of an attempt to adjust for confounding for the infection-telomere length analysis. | high | low | low | low | high | low | high |

Definitions: NI= Insufficient information, some = ‘some concerns’ relating to risk of bias, low= low risk of bias, high= high risk of bias, very high= very high risk of bias.
