# Supplementary material for: Infection and telomere length: A systematic review
Source: PLoS One. 2025 Sep 23;20(9):e0333107. doi: 10.1371/journal.pone.0333107 (PMC12456831; doi:10.1371/journal.pone.0333107)
Supplement: S3 Table — (DOCX) [file pone.0333107.s007.docx]

Table of 105 exposure-outcome relationships grouped by infection type, cell type, whether the outcome was TL or change in TL, TL measure (relative or absolute), TL measurement assay, and statistical method

| Infection [n E-O relationships] | Cell type group [n] | TL or change in TL [n E-O relationships] | TL measure [ E-O relationships ] | Measurement assay  [n E-O relationships ] | Statistical analysis method (effect estimate)  [n E-O relationships ] | Author, year | Country | Study design | Description of cell type | Sample size | Mean TL control vs exposed (IQR) [SD]  {SE} | Effect estimate (95% CI)  Exposed vs unexposed (ie. Unexposed is reference group) | Adjusted for | Evidence of association | Severity analysis | Age, sex stratification |
| --- | --- | --- | --- | --- | --- | --- | --- | --- | --- | --- | --- | --- | --- | --- | --- | --- |
| Hiv [35] | Non-specific  leukocytes including ‘leukocyte’, ‘peripheral leukocyte’, ‘serum leukocyte’, ‘leukocyte of whole blood’ [13] | TL [11] | Relative TL [10] | Q-PCR [10] | Linear regression (β coefficient) [5] | Mehta, 2021* | USA | Cross-sectional | Leukocyte | 161 | Not given | −0·034 (no CI given) | Age,sex, education, race/ethnicity , leukocyte count , METH Dependence, Body Mass Index, Serum Globulins, creatinine | Yes | N/A | N/A |
|  |  |  |  |  |  | Zanet, 2014*^a^ | Canada | Cross-sectional | Leukocyte | 395 | Median 3.0 (2.6–3.5) vs 2.9 (2.6–3.3) | −0.50 (−0.73 to −0.27) | ,Age (per 10 y),HCV infection status ,Smoking stat, HIV status × smoking status interaction, peak viral load hiv as binary variable high vs low Peak ie HIV pVL ≥100 000 copies/mL (vs <100 000 copies/mL | yes | Multivariable regression for viral load high vs low gave beta −.17 (−.34 to −.006) p=.04. | No evidence for interaction with age but HIV infection status was associated with shorter LTL for “younger subjects” only. Not clear which age group. |
|  |  |  |  |  |  | Macamo, 2024* | South Africa | Cross-sectional | Leukocyte | 100 | 1.21 ± [0.53] vs 0.96 ± [0.42] | -0.32 (-0.93 - 0.28)  Note: this was the fully-adjusted model, 3 other models with various levels adjustment showed an association between infection and reduced TL | Age, Gender, Body Mass Index (BMI), HIV status, Employment, Income source/Occupation, Monthly income, Education, Intake of alcohol per day, Intake of drugs or marijuana, Water source, Toilet type, Present diseases aware of, Any illness in the past 30 days, Previous worm infection, Deworming medication in the past 6 months, Allergic reaction in the past 30 days, Chronic illness, Medication taken, Supplements taken, Antibiotics/Probiotics taken in the past 2 weeks, Tuberculosis (TB) infected, Antiretroviral therapy,  Iron status markers, Serum minerals and proteins, Inflammatory and nutritional markers, Full blood count parameters. | No/ mixed | None | N/A |
|  |  |  |  |  |  | Yang, 2024^a^ | Canada | Cross-sectional | Leukocyte | 376 | Sex-stratified results only,   Females: (mean TL=8.8, 95% CI (8.5–9.1) vs 8.1 (7.8–8.3) p < 0.001)  Males: 8.3 (7.9–8.7) vs (7.9 (7.5–8.3) p=0.20 | -0.22 (-0.36- -0.09) | sex, 10 year age, ethnicity, smoking, total no. non-HIV viruses, virus type | yes | N/A | A difference between male and female groups was found but there was no test for interaction |
|  |  |  |  |  |  | Srinivasa, 2014* | USA | Cross-sectional | Leukocyte | 142 | Log mean 1.04 ± [0.05] vs 1.02 ± [0.04] | ‐0.19287 (no CI given) | Current smoking status (smoker) , Age (years) , sCD163 (ng/mL) | Yes/ weak | Among the HIV-infected cohort, only association between sCD163 and TL was “significant” (pearson correlation coefficient= -0.30, P = 0.003) whereas HIV-related parameters, including VL (viral load), CD4 count and duration ART use were not associated with TL | N/A |
|  |  |  |  |  | Linear regression (No β coefficient) [1] | Pathai, 2013* | South Africa | Cross-sectional | Leukocyte | 486 | 1.07 +-{0.008} vs 0.91+-{0.007} | Not provided | Unclear | Yes | No evidence for a difference between ART naïve and those on ART (p=0.71) there was evidence however for association of CD4+ count and reduced TL (P value test for trend= 0.02) | N/A |
|  |  |  |  |  | ANCOVA (non-standardised beta-coefficient) [1] | Von Kanel, 2014* | South Africa | Cross-sectional | Leukocyte | 341 | Not given | -0.054 ,standard error=0.024, p=0.023 (No CI given) | Unclear | Yes | N/A | N/A |
|  |  |  |  |  | ANCOVA (no effect estimate just mean TL) [2] | Imam, 2012^a^ | Canada | Cross-sectional | Leukocyte | 99 | 3.49 ± [0.78] vs 3.27 ± [0.74] | N/A | maternal age and smoking ever in pregnancy, maternal ethnicity, use of drugs of addiction,  methadone ever in pregnancy | No | N/A | N/A |
|  |  |  |  |  |  | Giesbrecht, 2014^a^ | Canada | Cross-sectional | Peripheral blood leukocyte | 126 | 2.9 [0.48] vs 2.9 [0.62] p= .62 | N/A | age | No | N/A | N/A |
|  |  |  |  |  | 2-way ANOVA (just mean TL) [1] | Malan-Muller, 2013* | South Africa | Cross-sectional | Leukocyte | 128 | 0.98 [0.36] vs 0.61 [0.2] | N/A | None/unclear | Yes | N/A | N/A |
|  |  |  |  |  | T-test or Mann Whitney (no effect estimate just mean TL) [1] | Toljic, 2023* | Serbia | Cross-sectional | Leukocytes of whole blood | 205 | HIV- <35 yrs 0.83 +- [0.36] vs HIV+ <35 yrs 0.87 +- [0.37] , HIV- >50 yrs 0.69 +- [0.24] vs HIV+ >50 yrs 0.79 +- [0.32] | N/A | None | no | No difference was found between TL’s of HIV+ and HIV- samples in either <35yr or >50yr old age groups |  |
|  |  |  | Absolute TL [1] | Q-PCR [1] | Linear regression (β coefficient) [1] | Liu, 2015* | Canada | Cross-sectional | Peripheral leukocytes | 922 | 150± {3} vs 123± {4} kbp/genome | Non-standardized β (SE) =10.406 (2.211), standardized β= -0.193, p<0.001 | age yrs, ever smoked, FEV1%Pred (forced expiratory volume in 1 second percent predicted) | yes | Within the HIV population only , significant predictors of shorter aTL in a multivariate linear regression model included having a nadir CD4 cell count 350/μL (p = 0.018) β (SE) -6.488 (2.726). Longer time from HIV diagnosis to enrollment, an estimate of the duration of HIV infection, was associated with the shortest aTL (p = 0.019). In addition, lower nadir CD4 cell counts were also associated with shorter aTL (p = 0.023), while plasma viral loads >100,000 copies/mL at the time of HIV | N/A |
|  |  | TL and change in TL ^1^[1] | Relative TL [1] | Q-PCR [1] | Linear regression (converted back into percent differences in LTL) [1] | Ding, 2018* | China | Cross-sectional | Leukocyte | 488 | 0.96 [0.40] vs 0.91[0.42] | -3.7%, (- 11.3% to 4.5%) P=0.367 | Gender, education level, and smoking history, insomnia and depressive symptoms | no | N/A | N/A |
|  |  | Unclear [1] | Relative TL [1] | Q-PCR [1] | MANOVA (β coefficient) [1] | Saberi, 2019^a^ | Canada | Cohort | Leukocyte | 105 | Not provided | -0.35 (-0.87 to 0.17 ) | Maternal age at delivery (years), Maternal age at delivery*Weeks of gestation, Weeks of gestation at Visit A, History of HCV infection a (yes vs. no), Smoking throughout pregnancy b (yes vs. no), Received PI/r during pregnancy (yes vs. no), Cohort (CARMA vs. Pregnancy) | no | N/A | N/A |
|  | PBMCs including ‘PBMCs’, ‘proliferative CD8+CD28+ T cells, senescent CD8+CD28- T cells, CD4+ T-cells’, ‘CD8+ enriched PBMCs’ and ‘lymphocytes (CD4, CD8, CD45RA, CD45RO)’ [14] | TL [11] | Relative TL [7] | Q-PCR [7] | Linear regression (β coefficient) [2] | Wang, 2019*^b^ | Uganda | Cross-sectional | PBMCs | 434 | N/A | -0.0497 (No CI given) | age, current smoking, pack-years, and asthma | yes | N/A | N/A |
|  |  |  |  |  |  | Auld, 2016^b^ | Uganda | Cross-sectional | PBMCs | 184 | median and iqr, 1.09 (0.9–1.2) vs1.02 (0.9–1.1) | -0.0621 (-0.113, -0.011) | gender, total pack years of cigarettes smoked, alcohol consumed in the past 12 months, and diagnosis of asthma | yes | N/A | N/A |
|  |  |  |  |  | Linear regression (No β coefficient*) [1] | Gogia, 2015* | USA | Cross-sectional | PBMCs | 89 | *Median* TL was 0.95 in treated and virally suppressed patients and 0.93 in untreated and non-suppressed, compared with 1.07 in uninfected controls (p=0.046), while median TL was 1.02 in untreated, virally suppressed patients (p=0.72 vs. controls). | Not provided | None | yes | See results column, also among HIV patients, viral load >10K copies/mL (p<0.001), was independently associated with shorter TL. | N/A |
|  |  |  |  |  | Spearman rank correlation (no effect estimate just median TL) | Petrara, 2024* | Italy | Cross-sectional | PBMCs | 78 | Median TL : healthy controls 1.3 (1.2-1.4) vs perinatally  acquired HIV 1.2 (1.1-1.3) , p=0.011 | Not provided | Age-matched | Yes | Perinatally acquired HIV was subgrouped into Not Suppressed (NS) with median TL 1.1 (1.1-1.2 IQR) p<0.001 vs healthy controls,  Early Suppressed (ES)1.3 (1.2-1.4) p=0.942 vs healthy controls and Late Suppressed (LS) 1.2 (1.1-1.3) p= 0.044 vs healthy controls | N/A |
|  |  |  |  |  | Unclear statistical method (no effect estimate just mean TL) [3] | Cobos Jimenez, 2016* | Netherlands | Cross-sectional | PBMCs | 189 | 0.55 ± [0.27] vs 0.40 ± [0.35] | N/A | Age-matched | Yes | N/A | N/A |
|  |  |  |  |  |  | Hsieh, 2015* | Unclear | Cross-sectional | PBMCs ( Proliferative CD8+CD28+ T cells, senescent CD8+CD28- T cells, CD4+ ) | See column right | Proliferative CD8+CD28+ T cells (n=27) : median 3.73 (3.48-4.08) vs 3.35 (2.63-3.93)  senescent CD8+CD28- T cells (n=29): 2.32 (2.15-2.84) vs 2.48 (2.18-3.16)  CD4+ T cells (n=26): 3.43 (2.96-3.57), vs 3.76 (3.21-4.14) | N/A | Age | Mixed | N/A | N/A |
|  |  |  |  |  |  | Gonzalez-Serna, 2017* | Canada | Retrospective cohort | PBMCs | 95 | *Median* Time 1 : 9.6 (IQR 8.7–11) vs 9.1 (IQR 7.8–11.1)  Time 2: 9.6 (IQR 8.8–11.2) vs 8.2 (IQR 6.9–10.0) | N/A | Age | Yes | N/A | N/A |
|  |  |  | relative telomere length compared to uninfected controls [1] | Q-PCR [1] | Analysed using Kruskal-Wallis followed by the Mann-Whitney U (No effect estimate just mean TL) [1] | Gaardbo, 2013* | Denmark | Cross-sectional | CD8+ enriched PBMC | 44 | *Median* telomere length in healthy controls (n = 12), controllers (n = 9), long term non-progressors (LTNP) (n = 11) and progressors (n = 12) were 1.09 (0.40-1.32), 0.39 (0.03-1.36), 0.55 (0.24-0.66) and 0.36 (0.15-0.89), respectively. Values are given relative to healthy controls. | N/A | Age, sex , ethnicity matched | Yes | Reduced TL in ‘progressors’ group vs LTNP (results column) | N/A |
|  |  |  | TRF length measured to calculate absolute TL [1] | Southern Blot [1] | Not clear (No effect estimate given) [1] | Tucker, 2000* | United Kingdom | Cross-sectional | lymphocytes (4 cell types) | 21 | (units here are Kilobase pairs) HIV-:  CD4: 6.6[ ±1.1] CD8: 6.6[ ±1.6], CD45RA: 7.4[ ±1.7], CD45RO: 5.8[ ±2.1]  HIV+: CD4: 7.5 ±{ [1.3], CD8: 7.4 ±{ [1.5], CD45RA: 7.8 ±{ [1.8], CD45RO: 7.3 ±{ [1.9] “no significant differences between groups” but doesn’t present p-values | N/A | None | No | Author states that there were no “significant” differences in the telomere lengths of T-cell subpopulations between HIV-infected non-progressors and HIV-infected (intermediate plus rapid) progressors, nor between treatment-naive individuals and those receiving antiretroviral therapy. No p-values presented | N/A |
|  |  |  | Absolute TRF length [1] | Southern Blot [1] | ANCOVA (No effect estimate) [1] | Richardson, 2000* | USA, France | Cross-sectional | PBMCs | 170 | mean TRF length (kbp) , control= 9.17± {0.19}, Slow/non-progressors (S/NP)= 7.59 ±{0.11}, Fast progressors (FP)= 7.25±{0.15} | ANCOVA Mean TRF, control= 9.12 ±{0.17}, S/NP = 7.64 ±{0.11}, FP= 7.29 ±{ {0.15} | Age | Yes | See results columns | N/A |
|  |  |  | DNAmTL [1] | Methylation [1] | T- test (No effect estimate) [1] | Shiau, 2024* | USA | Cross-sectional | PBMCs | 190 | Sample was within women only: 7.34 ± [0.23] vs 7.13 ± [0.31] vs , P < .001 | N/A | Sex (sample in women only) | yes | N/A | N/A |
|  |  |  | Not clear [1] | Q-PCR [1] | Linear regression (Beta coefficient)[1] | Babu, 2019* | India | Cross-sectional | PBMCs | 96 | Not provided | −2.84 ( −4.012 to −1.67) | Age, sex | yes | N/A | N/A |
|  |  | Change in TL/ rate of shortening [3] | DNAmTL  [2] | Methylation [2] | Linear regression (Beta coefficient)[1] | Sehl, 2021^c^ | USA | Cohort | PBMCs | 201 | The average rate-of-shortening in DNAmTL was significantly faster in HIV-infected men (−0.056 units per year of chronologic age vs. −0.019, p < 0.001 welch's tests), indicating a nearly 3fold increase in rate-of-shortening compared with uninfected men. | -0.035 (No CI given) p=0.000014 | Baseline estimated TL, race, ethnicity, tobacco, BMI, hepatitis B status, and HIV status, including all individuals. Also For100 ofthese seroconverters,matched seronegative controls were selected, matched on age (+/− 2 years), and hepatitis C virus (HCV) status | yes | No evidence that Cumulative plasma HIV viral load or Absolute CD4 T cell count were associated with DNAmTL | N/A |
|  |  |  |  |  | mixed effect model (F-value) [1] | Breen, 2022*^c^ | USA | Cohort | PBMCs | 204 | Visit A: non sero-converter age-adjusted DNAmTL (relative units)= 0.091 vs Sero-converter= 0.043, p=0.25 (t-test)  Visit b: Non sero-converter= 0.078 vs Sero-converter= -0.211^2^ p<0.001 (t-test) | F-value= 17.76 p<0.001) (F value, p value) | Age | Yes | N/A | N/A |
|  |  |  | Absolute TRF length [1] | Not clear [1] | Mann-Whitney U test (no effect estimate) [1] | Wolthers, 1996* | unclear | Longitudinal | PBMCs | 21 | mean and SD loss of TRF length of 114 +- 100 bp/year  in the group of eight asymptomatics (hiv+ve) and  175 +- 105 bp/year in the group of six  progressors (hiv+ve) compared with 4.7 +- 71 bp/year  in healthy controls (n=7) (P = 0.04 and P =  0.008 respectively,). | N/A | Age-matched | Yes | See mean TL column | N/A |
|  | Non-specific blood cells  [4] | TL [3] | DNAm-TL [1] | Methylation [1] | Linear regression (Beta coefficient) [1] | Shiau, 2021* | USA | Cross-sectional | Not clear- says blood extracted | 107 | 7.07 ± [0.20] vs 6.77 ± [0.35] P < .001 compared between groups using t tests | β = −0.26, ( −0.41 to −0.12) | Tobacco use,and BMI. | yes | N/A | N/A |
|  |  |  | Relative TL [2] | Q-PCR [2] | Kruskall-Wallis test for comparison between the PWH, blood donor, and general population (no effect estimate) [1] | Cadinanos, 2024* | Spain | Cross-sectional | “Blood telomere length” | 384 | median HIV+ group: 1.07 (0.95–1.17) vs HIV-blood donors 1.28 (1.12–1.48) p<0.001  HIV- general population group >60 years .89 (IQR, 0.77–0.98); p<0.001 when compared with HIV+ group) | N/A | Age and sex matched (blood donor group only) | yes | N/A | N/A |
|  |  |  |  |  | No effect estimate just mean TL [1] | Woods, 2023* | USA | Cross-sectional | Not clear/ states “blood” | 149 | 0.9 [0.3] vs 0.9 [0.3] p=0.6284 | N/A | None | No | N/A | N/A |
|  |  | TL and change in TL* [1] | Relative TL [1] | Q-PCR [1] | linear regression(beta coefficient)[1] | Womersley, 2021* | South Africa | cross-sectional and longitudinal | Not clear just says DNA was extracted from whole blood | 286 (cross-sectional), 110 (longitudinal) 1 year follow-up | baseline TL :1.19 [0.46] vs 0.93 [0.29] t-test p< 0.001  One year mean: 0.96 [0.25] vs 0.99 [0.29] t test p=0.603 | Cross-sectional:β = −0.26, t p < 0.001(no CI given)  Longitudinal results relating to the association of HIV infection and telomere length could not be extracted | None but tested associations | yes | N/A | N/A |
|  | PBMCS + whole blood [1] | TL [1] | DNAm-TL [1] | Methylation [1] | linear regression(beta coefficient)[1] | Liang, 2024* | USA | Cross-sectional | PBMCS + whole blood [1] | Veterans Aging Cohort Study 1(VACS1, N= 1251)  Women's Interagency HIV Study Cohort  (WIHS, N= 481) | Not available | VACS1, β= −0.25 (−0.32, −0.18)   WIHS , β=−0.01 (−0.08, 0.05) | Age, self-reported race and ethnicity, BMI, assay sample  batch, smoking status, alcohol consumption , cell type | Yes and no | N/A | N/A |
|  | peripheral blood osteogenic precursor (COP) cells (LIN−/OCN+) [1] | TL [1] | Relative TL [1] | Q-PCR [1] | Wilcoxon signed-rank test (No effect estimate) [1] | Manavalan, 2016* | USA | Cross-sectional | peripheral blood osteogenic precursor (COP) cells (LIN−/OCN+) | 45 | telomere lengths of LIN−/OCN+ cells were shorter in the perinatally-infected group (n = 15, T/S ratio median (interquartile range) of 2.201 (2.064 to 2.459) compared to adolescence-infected (n = 15, T/S ratio median 2.523 (2.463 to 2.569), p = 0.003 or uninfected controls (n = 15, T/S ratio median 2.546 (2.275 to 2.692) p = 0.018. | N/A | None | Yes | N/A | N/A |
| Covid-19 [10] | Non-specific  leukocytes including ‘leukocyte’, ‘peripheral blood leukocytes’ [6] | TL [6] | Relative TL [4] | Q-PCR [4] | Inverse variance weighted with random effects (beta coefficient) [2] | Huang, 2022^d^ | European cohorts | Mendelian randomisation study | Non-specific leukocyte | 1,388,342 for genome-wide association studies of critically ill COVID-19 patients and 472,174 for studies of LTL | N/A | β =0.0075, (−0.018 to 0.021) p=0.733) | N/A | No | N/A, although this was measuring ‘critically-ill’ covid-19 but no ‘non-critically ill’ covid-19 for comparison | N/A |
|  |  |  |  |  |  | Xu, 2022^d^ | European cohorts | Mendelian randomisation study | Non-specific leukocyte | telomere length: 472174. Covid susceptibility: (1,683,768 total).Covid severity: (1,388,342 total) | N/A | Susceptibility:0.02 (0.0004, 0.05) P = 0.05673 severity: −0.01 (−0.02, −0.001P = 0.02779 | N/A | Susceptibility no, severity yes. Reverse relationship also no. | See effect estimate column | N/A |
|  |  |  |  |  | Inverse variance weighted with fixed effects (beta coefficient) [1] | Jiang, 2022*^d^ | European cohorts | Mendelian randomisation study | Non-specific leukocyte | LTL: 78,592  Covid-19susceptibility: covid positive (N= 38,984 )versus population controls (N = 1,644,784)  Covid-19 severity: exposed  (N = 9,986) versus  population controls (N = 1,877,672) | N/A | Susceptibility C2: -0.022 (-0.11 to 0.07)p=0.630,  Severity B2: -0.001 (-0.04 to 0.04) p=0.949  Severity A2: 0.009 (-0.02 to 0.04)p=0.593 | N/A | No, in neither direction | See effect estimate column | N/A |
|  |  |  |  |  | Mann–Whitney U test (no effect estimate just median) [1] | Krasnienkov ,2022* | Ukraine | Cross-sectional | Peripheral blood leukocytes | 106 | Median 0.46 (0.19-1.03) vs 1.49 (0.80-2.55), (p< 0.01) | N/A | Sex (study in women only), however not matched or adjusted | yes | N/A | N/A |
|  |  |  | Absolute TL [2] | TeSLA and Southern Blot [1] | Not clear [1] | Benetos, 2021* | France | Cross-sectional ^3^ | Non-specific leukocyte | 38 | Mean from TeSLA :3.53 kb ± [0.39] vs 3.42 ±[0.33] p=0.37  SB: 6.64 ± [0.58] vs 6.52 ± [0.56] p=0.35 | N/A | none | No | N/A | N/A |
|  |  |  |  | Q-PCR [1] | Absolute (Kb) or age-adjusted (Z-score) TL was compared in the diferent groups by non-parametric Mann Whitney or ANOVA (Kruskal-Wallis) tests (no effect estimate) [1] | Retuerto, 2022* | Spain | Cross-sectional | Peripheral blood leukocytes | 420 | Healthy control Z-score: 0.19 (− 0.66, 0.44) vs hospitalized COVID-19: − 1.45 (− 2.64, − 0.12) p < 0.0001 | N/A | Age standardised, | Yes, this study was looking at reverse relationship but is cross-sectional so can’t distinguish | There was in analysis but the analysis relevant to this systematic review. | N/A |
|  | Granulosa Cells [1] | TL [1] | Absolute TL [1] | qFISH [1] | t-test (no effect estimate just mean TL) [1] | Chico-Sordo, 2022* | Spain | Cross-sectional | Granulosa Cells [1] | 65 | Non covid (units unclear) :141.4 ± 67.5 SD, COVID: 143.2 ± 72.9 | N/A | none | No, severe vs controls yes | Mean TL of GCs from severe COVID-19 cases 96.1 ± 21.9 was  statistically significantly lower (P = 0.017) compared to controls 143.2 | N/A |
|  | Un-specified peripheral blood cells [1] | TL [1] | Absolute TL [1] | Q-PCR [1] | Two sided t-test (no effect estimate just mean TL) [1] | Mongelli, 2021* | Unclear | cross-sectional | Un-specified peripheral blood cells | 261 | No covid (kb): 10.67 ± [11.69] , post covid: 3.03 ± [2.39], p<0.0001 (two-sided t-test) | None | Age- and sex-matched | Yes | N/A | N/A |
|  | Un-specified blood cells [1] | TL [1] | Relative TL [1] | Q-PCR [1] | Mann-Whitney U test (no effect estimate just mean TL) [1] | Savrun, 2023* | Turkey | cross-sectional | Un-specified blood cells | 140 | 1.26±[0.76] vs 0.93±[0.58]  P=0.0463 | None | None | Yes | “Mortality and malignancy had no statistically  significant correlation with short telomere length (p=  0.567 and p = 0.158, respectively)” | Female patients with COVID-19 (+) (mean±std:0.76±0.54) had shorter telomere lengths than male patients (mean±std:1.06±0.50) (p = 0.0282). No interaction with age found. |
|  | Sperm cells [1] | TL [1} | Relative TL [1] | Q-PCR [1] | MANOVA ( F-value as the ratio of two mean square values ) | Soares, 2025* | Brazil | Cross-sectional | Sperm cells | 112 | 4.69±[1.89] vs 4.06±[1.89] , t-test p-value= 0.041 | F= 4.30, p= 0.0406 | Sex (as in men only), age, any comorbidity, physical activity | yes | N/A | No evidence for interaction with age was found. |
| CMV [8] | Leukocyte non-specific and specific leukocytes [6] | ‘TL’ or ‘TL + change in TL’ [6] | ‘Relative TL’ or ‘log relative TL’ [4] | Q-PCR [4] | Linear regression (Beta coefficient) [4] | Aiello, 2017* | USA | Cross-sectional | Non-specific leukocyte | 163 | Mean difference in TL between unexposed group and exposed group −0·0267 {0·0352} p=0·449 | −0·0377 (0·04) p=0·323 | age, race/ethnicity, sex, education level, pack-years of smoking, BMI (kg/m2), and diabetes history | No | N/A | CMV seropositivity was significantly associated with shorter telomere length among females (β=−0·1204 (standard error (S.E.) 0·06), P= 0·044) in linear regression fully adjusted model. However there was only weak evidence for an interaction with sex (p=0.08) |
|  |  |  |  |  |  | Yang, 2024* | Canada | Cross-sectional | Leukocyte | 376 | N/A | 0.01 (-0.16-0.18) | sex, 10 year age,HIV status, ethnicity, smoking, total no. non-HIV viruses, virus type | no | N/A | N/A |
|  |  |  |  |  |  | Noppert, 2020* | USA | Cross-sectional | Non-specific leukocyte | 1708 | Not provided | 0.006 (-0.03, 0.04) ,p=0.68 | age, sex, race, education, BMI, cigarette smoking status, CRP, white blood cell count | No | N/A | No evidence for interaction with age or sex was found. |
|  |  |  |  |  |  | Dowd, 2017*^e^ | United Kingdom | Cohort and cross-sectional | Non-specific leukocyte | 400 | Not provided | follow-up LTL (ie cross-sectional): beta (SE) −0.059 (0.014) p<.001 , change in LTL (ie longitudinal): -.057 (.015 SE) p<.001 (95% CI -.0857611 to -.0287036). | age, sex, smoking, employment grade, categorical body mass index, and baseline LTL and mutually adjusted for the other infections | yes | Higher CMV IgG antibody levels (mean, 3.01 OD; SD, 0.77) were significantly associated with greater LTL attrition (β = −0.029 [SE, 0.013]; P = .03). | “ No significant sex-infection interactions were found” |
|  |  |  | Absolute TL [2] | flow-FISH [2] | unpaired t-test (No effect estimate) [1] | Spyridopoulos, 2009* | Unclear | cross-sectional | Various leukocyte cell types | 13 in CHD patients, 20 I health individuals | “Healthy control subjects did not show any difference in TL from lymphocyte subsets between seropositive and seronegative subjects”,  CMV positive CHD patients had significantly shorter telomeres in their CD8+ T cells (5.18 +-0.99 versus 6.00+-0.55 kb, P=0.025) and CD8+CD28- cytotoxic T cells (4.37+-0.98 versus 5.52+-0.57 kb, P=0.006; but not in other lymphocyte populations, including CD8+CD28+ cytotoxic T cells | N/A | none | Mixed- only association within CHD patients. | N/A | N/A |
|  |  |  |  |  | Ordinary least squares regression ( Ordinary least squares effect) [1] | Andreu-Sanchez, 2024* | Netherlands | Cross-sectional | Lymphocytes, Granulocytes, Naive T-cells, Memory T-cells, B-cells, NK-cells | 1,243 | N/A | Ordinary least squares effect (standard error, p-value)  Lymphocytes: -0.29 (0.05, p = 3.27 × 10⁻⁸)  Granulocytes: -0.12 (0.06, p = 0.04) Naive T-cells:, -0.17 (0.05, p = 1.18 × 10⁻³)Memory T-cells: -0.23 (0.06, p = 6.43 × 10⁻⁵) , B-cells: -0.13 (0.06, p = 0.02), NK-cells: -0.53 (0.05, p = 3.09 × 10⁻²²) | Unclear | Yes | N/A | “in a subsequent CMV-sex interaction analysis, we observed a significant decrease of TL in NK-cells/fully differentiated T-cells  of women infected with CMV as compared to men (ols, effect = 0.28, p = 7.6x10^3  )” |
|  | ‘PBMCs’, ‘CD4+T cells and CD8+ T cells’ [2] | TL [2] | Relative TL [2] | flow-FISH [1] | Mann– Whitney U-test (No effect estimate)[1] | Meijers, 2013* | Unclear | cross-sectional | CD4+T cells and CD8+ T cells | 159 | Values for most comparisons not reported as ‘non-significant’. One numeric result reported : “young seropositive end-stage renal disease patients had significantly (P < 0·05) shorter telomeres within their CD8+ T cell compartment (mean RTL ± s.e.m.; 11·19 ± 0·83%) when compared to CMV-seronegative age-matched counterparts (13·28 ± 0·75%)” | N/A | Age and sex matched | Mixed results, Mostly no except in CD8+ cells in young ESRD patients. | N/A | N/A |
|  |  |  |  | Q-PCR [1] | Linear regression (Beta coefficient) [1] | Dowd, 2013^e^ | United Kingdom | cross-sectional | PBMCs | 434 | Not provided | CMV seropositivity (β=−0.003, p=.674) was not associated with leukocyte TL | Age, sex | no | CMV IgG (within CMV+ participants only) (β= −0.005, p=0.45) was not associated with leukocyte TL | CMV + within men beta:−0.011 p=0.318, within women:0.004 p=0.665, no evidence of interaction with sex. Study also states there was no interaction with age. |
| H. Pylori [6] | Non-specific leukocyte [4] | TL [4] | ‘Relative TL’ or  ‘Log relative TL’ [3] | Q-PCR [3] | Linear regression (Beta coefficient)[3] | Aiello, 2017* | USA | cross-sectional | Leukocyte non-specific | 163 | Mean difference in TL from unexposed reference group 0·0087 {0·0269} p=0·747 | −0·0108 (0·03 SE) p=0.706 | age, race/ethnicity, sex, education level, pack-years of smoking, BMI (kg/m2), and diabetes history. | No | N/A | No interaction with sex found |
|  |  |  |  |  |  | Huang, 2020*^f^ | USA | cross-sectional | Leukocyte non-specific | 3472 | units (base pairs^4^, standard error) H.Pylori-: 5794 {50.6} vs H.Pylori +:5753 {53.5} | 39.7 (−46.6, 125.9) | age (year), sex (male, female), race (Hispanic, non-Hispanic white/black, other), education (<9th, 9th-11th grade, GED, some college, college, or higher), family income (quartiles), marriage (married, never married, other), place of birth (United States, other countries), smoking status (never, ever, current), and BMI categories (normal, overweight, obese); CRP (mg/dL), comorbidity of cardiovascular, respiratory diseases, hypertension, hypercholesterolemia (yes, no), and ulcer (yes, no) | No, except for in elderly | N/A | N/A |
|  |  |  |  |  |  | Noppert, 2020^f^ | USA | cross-sectional | Leukocyte non-specific | 1708 | Not provided | 0.01 (-0.03, 0.05) p=0.51 | age, gender, race, education, BMI, cigarette smoking status, CRP, white blood cell count | No | N/A | No evidence for interaction with age or sex was found. |
|  |  |  | Absolute TL [1] | Southern blot [1] | Linear regression (Beta coefficient) [1] | Muhsen, 2019* | Israel | cross-sectional | Leukocyte non-specific | 934 | 6.65 kb [0.66] vs 6.78 kb [0.59], p = 0.016 (t-test) | Presenting the results for their second model adjusted for more variables, H. pylori negative, no atrophic gastritis= Reference. H. pylori positive, no atrophic gastritis 0.07 (-0.05, 0.18) p=0.2. H. pylori positive plus atrophic gastritis 0.18 (-0.02, 0.37) 0.08. Past H. pylori infection (IgG sero-negative) plus  atrophic gastritis  -0.19 (-0.40, 0.01) 0.06. Note that their 1^st^ model adjusted for less variables, the relationship for past H. pylori infection (IgG sero-negative) plus  atrophic gastritis  gave a p-value of 0.049 | age, sex, religiosity, education, marital status, number of siblings, smoking, obesity, and physical activity | No , or weak association with increased TL | See adjusted results column | No significant interactions were found between H. pylori-atrophic gastritis sero-status with sex (p = 0.2), age (p = 0.12) |
|  | Gastric mucosa cells [2] | TL [2] | Relative TL [2] | Q-PCR [2] | Unclear (No effect estimate) [1] | Tahara, 2013*^g^ | Unclear | cross-sectional | Gastric mucosa cells | 150 | 4.03±[1.96] vs. 2.82±[1.62] p=0.002 | N/A | None | Yes | There was assessment of degree of gastritis but this can have other causes so was not included | N/A |
|  |  |  |  |  | Student-t test (No effect estimate) [1] | Yoshioka, 2012^g^ | Japan | cross-sectional | Gastric mucosa cells | 150 | 3.55 ± [0.27] vs. 2.97 ± [0.18], p = 0.07 | N/A | None | Weak association | N/A | N/A |
| HCV [6] | PBMCs including ‘PBMCs’ ‘T-cells’ [3] | TL and Change in TL [1] | Relative TL [1] | Q-PCR [1] | Mann–Whitney or analysis of covariance tests (No effect estimate) [1] | Gonzalez-Serna, 2017* | Canada | Retrospective cohort | PBMCs (non-specific) | 95 | After adjusting for age, there were no differences in median telomere length at T1 between participants in the 3 groups [HCV: 8.5 (IQR 6.9–10.0) vs. HIV: 9.1 (IQR 7.8–11.1) vs. Controls: 9.6 (IQR 8.7–11)] . At T2, telomere length was shorter in both seroconverter groups compared with controls [HCV: 8.4 (IQR 7.2–9.9) and HIV: 8.2 (IQR 6.9–10.0) vs. Controls: 9.6 (IQR 8.8–11.2), P = 0.02 and P = 0.01, respectively] after adjusting for age. | N/A | age | Yes | N/A | N/A |
|  |  | TL [1] | Relative TL [1] | Q-PCR [1] | None [1] | Hartling, 2013 * | Denmark | Cross-sectional | PBMCs (non-specific) | 75 | (mean with 95% CI) healthy controls 1.0 (0.75–1.25). HCV with fibrosis 0.88 (0.62–1.14) HCV without fibrosis 1.15 (0.90–1.39) | N/A | None | No | N/A | N/A |
|  |  | Change in TL [1] | median telomere length of T cell subsets relative to telomere length of calf thymocytes [1] | FLOW-FISH [1] | Unclear (No effect estimate) [1] | Grady, 2013* | Netherlands | Cohort | CD8+ and CD4+ T-cells | N=74,  4 groups including 1. healthy individuals (n = 22) ; 2. Drug-users who were Multiple Exposed (to HCV) Uninfected (MEU) (n = 8);  3. chronic HCV (cHCV) monoinfected drug-users (n =21); 4. cHCV/HIV coinfected drug-users (n = 23). | CD8+ T-cells  Telomere length within Cd8+ T-cells decreased from a median RTL of (0.36 (0.24–0.33 IQR) to 0.28 ( 0.24– 0.33) in MEU, 0.36 (IQR 0.32–0.41) to 0.30 (IQR 0.26–0.34) in cHCV monoinfected and 0.30 (0.27–0.30) to 0.23 (0.21–0.28) in cHCV/HIV coinfected drug-users. There was no ‘significant’ difference between multiple exposed uninfected or healthy individuals and the hcv group. Note they did not provide the full results of the healthy individuals which is why it is not displayed here.  CD4+ T-cells  The study does not present the HCV mono-infected results for CD4+ T-cells. | N/A | None | No | N/A | N/A |
|  | Leukocyte non-specific [3] | TL [3] | Relative TL [3] | Q-PCR [3] | Linear regression (Beta coefficient) [2] | Zanet, 2014*^h^ | Canada | Cross-sectional | Leukocyte | 395 | Not provided | Active HCV infection (vs never) −.19 (−.35 to −.03) p=.02. Cleared HCV infection (vs never) −.16 (−.37 to .04)p=0.12 | HIV status (infected vs uninfected) ,Age (per 10 y),HCV infection status ,Smoking stat, HIV status × smoking status interaction, peak viral load hiv as binary variable high vs low Peak ie HIV pVL ≥100 000 copies/mL (vs <100 000 copies/mL | Yes | N/A | N/A |
|  |  |  |  |  |  | Yang, 2024^h^ | Canada | Cross-sectional | Leukocyte | 376 | N/A | -0.02 (-0.28-0.23) | sex, 10 year age,HIV status, ethnicity, smoking, total no. non-HIV viruses, virus type | no | N/A | N/A |
|  |  |  |  |  | MANOVA (β coefficient) [1] | Saberi, 2019^h^ | Canada | Cohort | Leukocyte | 105 | Not provided | history HCV: 0.03 (-0.70 – 0.77) p= 0.93 | Maternal age at delivery (years), Maternal age at delivery*Weeks of gestation, Weeks of gestation at Visit A, HIV (yes vs. no), Smoking throughout pregnancy b (yes vs. no), Received PI/r during pregnancy (yes vs. no), Cohort (CARMA vs. Pregnancy) | No | N/A | N/A |
| Hbv [5] | ‘leukocytes’ , ‘peripheral blood leukocytes’ [3] | TL [1] | ‘Relative TL’ or ‘log relative TL’ [3] | Q-PCR [3] | Linear regression (Beta coefficient) [2] | Noppert, 2020* | USA | cross-sectional | Leukocyte non-specific | 1708 | Not provided | -0.02 (-0.05, 0.01) p=0.28 | age, gender, race, education, BMI, cigarette smoking status, CRP, white blood cell count | No | N/A | No evidence for interaction with age or sex was found. |
|  |  |  |  |  |  | Zanet, 2014* | Canada | Cross-sectional | Leukocyte | 395 | Not provided | -0.30 (-0.57 to -0.02) P=.04 | none | Yes but unadjusted | N/A | N/A |
|  |  |  |  |  | Logistic regression (Odds ratio) [1] | Ma, 2016* | China | Cross-sectional | peripheral blood leukocytes | 396 | [RTL median (range)] control 1.82 (0.33–7.67) , CHB 2.07 (0.62–7.31), p=0.002 Student t test | when control was used as the reference  group and RTL was dichotomized into long and short groups  based on the median value in control group, individuals with  longer RTL had an increased risk of CHB (OR: 1.83, 95% CI: 1.22–2.73). | none | Reverse relationship ie hbv associated with long TL. | N/A | No evidence/ test for interaction with age or sex but median relative LTL varied by age and gender… CHB male 2.00 (0.62–7.31) vs control male 1.78 (0.53–3.63) p=0.023. CHB female 2.13 (0.85–4.29) vs 1.87 (0.33–7.67) p=0.038. age <50 CHB 2.06 (0.62–7.31) vs control 1.88 (0.33–7.67) p=0.099. age>or equal to 50, CHB 2.14 (0.83–3.81) vs 1.69 (0.55–3.41) p=0.004 |
|  | Hepatocytes [1] | TL [1] | Mean fluorescent intensity (MFI) [1] | Q-FISH [1] | Unclear [1] | Tachtatzis, 2011* | Unclear | Cross-sectional | hepatocytes | 94 | Hepatocyte telomeres (mean) were longer in controls than HBV carriers (467.8 vs 125.6, p<0.0001) | N/A | none | Yes but very high ROB and no adjustment for confounding | N/A | N/A |
|  | PBMCs [1] | Change in TL/ rate of shortening [1] | DNAmTL  [1] | methylation [1] | Linear regression (Beta coefficient)[1] | Sehl, 2021* | USA | Cohort | PBMCs | 201 | Not provided | −0.0074 p=0.80 | Unclear | No | N/A | N/A |
| Hpv [2] | cervical epithelial cells [1] | TL [1] | Relative TL [1] | Q-PCR [1] | Student’s t-test (No effect estimate) [1] | Albosale, 2021* | Unclear | Cross-sectional | cervical epithelial cells | 100 | 1571.21 ± {111.55} vs 1144.06 ± {64.085} vs, p=0.0014 | N/A | none | Yes | HPV was categorized into two groups based on viral load: 4-5 lg and >5 lg HPV genomes per 100,000 human cells. The T/S ratio (Mean ± SE) was 1571.21 ± 111.55 in the HPV-negative control group, 1035.12 ± 65.93 in the 4-5 lg group (p=0.0001 vs control), and 1222.93 ± 98.05 in the >5 lg group (p=0.0217 vs control), with no significant difference between the two HPV load groups. | N/A |
|  | peripheral blood leukocytes [1] | TL [1] | Relative TL [1] | Q-PCR [1] | Unclear [1] | Hampras, 2016* | USA | Cross-sectional | peripheral blood leukocytes | 336 | 1.16 [0.57] vs 1.24[0.75], P = 0.56 | N/A | None for this analysis | No | N/A | N/A |
| High-risk hpv [4] | esophageal squamous cell carcinoma (ESCC) and paired matched adjacent noncancerous tissues [1] | TL [1] | Relative TL [1] | Q-PCR [1] | Mann-Whitney 2-sample U test (no effect estimate) [1] | Zhang, 2014* | China | Cross-sectional | esophageal squamous cell carcinoma (ESCC) and paired matched adjacent noncancerous tissues | 70 | In tumour tissue: hpv- 0.62 (0.18-0.67) vs hpv+ 0.79 95% CI (0.48-0.89) vs p<0.05. In Non-tumour tissue: 0.61 (0.23-1.03) vs 0.74 (0.25-1.00) p > 0.05 | N/A | None | No (longer TL in exposed) | >1 HR-HPV (copies/celll was associated with longer telomere length than <1 copies/cell in tumour tissue but no difference was found between these in non-tumorous tissue | N/A |
|  | Non-specific blood and cervical cells [1] | TL [1] | Absolute TL [1] | Q-PCR [1] | None [1] | Panczyszyn, 2020* | Unclear | Cross-sectional | Non-specific blood and cervical cells | 88 | Women from the control group had longer telomeres in cervical  smears compared to the lesion+hrhpv group (average telomere length:  533 ± [169] vs 401 ± [158] kbp/genome) and the hrhpv only group  (437 ± [169] kbp/genome). “There was no statistically significant  difference between the case group and the carrier group in telomere length of cervical smears”. The length of telomeres from women blood was analyzed. The analysis included 21 women from the case group, 14 women from the carrier group, and 8 women from the control group. The average telomere length in blood in all study groups was similar case group: 384 ± [162], carrier group: 387 ± [87], control group: 408 ± [149] kbp/genome) | N/A | None | Maybe- No statistical analysis | N/A | N/A |
|  | Human cervical epithelial cells [2] | TL [2] | Relative TL [2] | Q-PCR [2] | T-test (no effect estimate) [2] | Al-Awadhi, 2023* | Kuwait | Cross-sectional | Human cervical epithelial cells | 287 | Hpv- controls: 1.28 ± [1.06], hpv+:3.42 ± [2.38] , hpv+ and squamous intraepithelial lesion: 3.31 ± [3.00]  RTL in both hpv+ groups was larger than in controls, p<0.001 | N/A | None | Opposite association/ infection associated with longer TL | N/A | N/A |
|  |  |  |  |  |  | Wang, 2022* | China | Cross-sectional | Human cervical epithelial cells | 1318 | 1.30 ± [0.60], 1.19 ± [0.48] ,P < 0.001 (student t-test) | N/A | None | Yes | When divided into HPV16/18 and other hrHPV infected groups, telomere length was 1.17 ± 0.47 and 1.19 ± 0.48, respectively. There is no difference in telomere length between two HPV-infected groups (P = 0.522), however, telomere length in either hrHPV group was significantly shorter than that in the non-infected group (other hrHPV or HPV16/18 vs non-infected: P < 0.001) | N/A |
| HSV-1 [4] | Leukocyte non-specific [4] | ‘TL’ or ‘TL + change in TL’ [4] | ‘Relative TL’ or ‘log relative TL’ [4] | Q-PCR [4] | Linear regression (Beta coefficient) [4] | Aiello, 2017* | USA | cross-sectional | Leukocyte non-specific | 163 | Mean difference in TL (Standard error) from unexposed reference group −0·0095 {0·0461} p=0·836 | 0·0008 (0·05 SE) p=0·987 | Adjusted for age, race/ethnicity, sex, education level, pack-years of smoking, BMI (kg/m2), and diabetes history. | No | N/A | No interaction with sex found |
|  |  |  |  |  |  | Dowd, 2017* | United Kingdom | Cohort and cross-sectional | Non-specific leukocyte | 400 | Not provided | follow-up LTL (ie cross-sectional): beta (SE) −0.047 (0.017) p<0.006 , change in LTL (ie longitudinal): -.047 (0.018 SE) p=0.008 (95% CI -0.0815111 -0.0124211) | age, sex, smoking, employment grade, categorical body mass index, and baseline LTL and mutually adjusted for the other infections | yes | No ‘significant’ associations were found for HSV-1 (β = 0.085 [0.075]; P = .26) for IgG levels and telomere length or attrition | “ No significant sex-infection interactions were found” |
|  |  |  |  |  |  | Yang, 2024* | Canada | Cross-sectional | Leukocyte | 376 | N/A | -0.07 (-0.23-0.10) | sex, 10 year age, HIV status, ethnicity, smoking, total no. non-HIV viruses, virus type | No | N/A | N/A |
|  |  |  |  |  |  | Noppert, 2020* | USA | cross-sectional | Leukocyte non-specific | 1708 | Not provided | 0.002 (-0.03, 0.03) p0=.90 | age, gender, race, education, BMI, cigarette smoking status, CRP, white blood cell count | No | N/A | No evidence for interaction with age or sex was found. |
| TB [2] | PBMCs [2] | TL [2] | Relative TL [2] | Q-PCR [2] | Linear regression (Beta coefficient) [1] | Wang, 2019*^i^ | Uganda | Cross-sectional | PBMCs | 434 | Not provided | 0.0015 p=0.95 | None | No | N/A | N/A |
|  |  |  |  |  | Logistic regression (Odds ratio) [1] | Auld, 2016^i^ | Uganda | Cross-sectional | PBMCs | 184 | Not provided | Odds Ratio (95% CI) 0.425 (0.059, 2.960) , p=0.39 | measures of pneumonia severity (oxygen saturation measured  while breathing room air), and overall health (ambulatory status) | No | N/A | N/A |
| Multi-drug resistant TB [1] | Peripheral blood cells [1] | TL [1] | Relative TL [1] | Q-PCR [1] | ANCOVA (standardized coefficient) [1] | Freimane, 2021* | Latvia | Cross-sectional | Peripheral blood cells | 108 | 1.022 ± [0.332] ,MDR-TB patients: 0.777 ± 0.294, | standardized coefficient ± SD where y MDR TB is reference group: 0.321 ± 0.102 (0.119 to 0.523), p= 0.002 | None/ loosely age and sex matched | Yes | patients with pulmonary TB had longer telomeres than those with extrapulmonary TB or extrapulmonary TB in combination with pulmonary TB; “however, these differences were not statistically significant” | 2-ΔΔCT and SD TB+males 0.746 ± 0.295 TB+females 0.879 ± 0.275 TB-males 1.038 ± 0.293 TB-females 0.971 ± 0.449. No statistical test performed. |
| Periodontitis [2] | Leukocyte [2] | TL [2] | Absolute + relative TL [2] | Q-PCR [2] | Unclear (No effect estimate) [1] | Nguyen, 2022^j^ | USA | Cross-sectional | Leukocyte | 3,454 | Men: weighted mean bp no peri : 5766+-[602] vs peri 5715 +-[569]    Women: 5828+- [630] vs 5820+-[647]  Among study subjects who received  periodontal assessment, telomere lengths  were significantly different between those who had and didn’t have periodontitis (P < 0.001)- unclear which statistical test used. | N/A | None | Yes | N/A | N/A |
|  |  |  |  |  | Linear and logistic regression (beta coefficient, odds ratio) [1] | Song, 2020*^j^ | USA | Cross-sectional | Leukocyte | 3,478 | base pairs mean (SE), no periodontitis: 5,782 {35}, mild:5,718 {49}, moderate: 5,676 {57} , severe 5,760 {131} | Linear regression results  Beta (SE) –46.0 (31.9)  Unexposed is reference group and mild to severe periodontitis is outcome “ p not less than 0.05”  Logistic regression results  Where ‘none’ (referring to periodontitis) is the reference group, mild-severe was the exposed group.  The odds of short telomere length in the exposed group was= 1.21 (0.96 to 1.53)  Where none/mild is reference ground vs Moderate to severe then the result is ‘statically significant’ odds of short telomere length is 1.38 (1.01 to 1.90) times greater in the exposed vs unexposed group. | age (year) and age quadratic (year2), sex (male, female) and race/ethnicity (Hispanic, non-Hispanic Black, non-Hispanic White, and others); family income to poverty ratio (quartile groups), education (<9th grade, 9th to 11th grade, high school graduate/GED, some college, college graduate or above), smoking (never, former, current), BMI groups (undernutrition, normal, overweight, obese); and cardiometabolic comorbidity (yes, no). | No, only when data is re-categorised an association is detected. | Logistic regression analysis was further separated to compare the reference group of ‘no periodontitis’ vs ‘mild, moderate and severe periodontitis separately Only moderate vs none provided evidence for an association with shorter TL OR (95% CI) 1.47 (1.04 to 2.09) | There was no evidence to suggest an interaction with sex,but compared with those with mild or no periodontitis, moderate and severe periodontitis was significantly associated with short-TL in those who were female (adjusted OR, 1.76; 95% CI, 1.10 to 2.83. This association was not found when comparing ‘any periodontitis’ with ‘no periodontitis’. No interaction was found with age group. |
| Sepsis [3] | Leukocyte [2] | TL [2] | Unclear [2] | Q-PCR [2] | random-effects inverse-variance weighted estimation method (Beta coefficient) [2] | Jiang, 2023 ^k^ | United Kingdom | bidirectional Mendelian randomization (MR) study | Leukocyte non-specific | GWAS data of leucocyte telomere length were all  obtained from a European population of 472,174  participants.  GWAS data for both sepsis and septic 28-day all cause death were obtained from the same European  population, including 486,484 participants | N/A | (sepsis to LTL),: β (SE) −0.0051 (0.0075) p=0.499. | N/A | Reverse, short telomere increases susceptibility to sepsis | N/A | N/A |
|  |  |  |  |  |  | Xu, 2024*^k^ | United Kingdom | bidirectional Mendelian randomization (MR) study | Leukocyte non-specific | GWAS data of leucocyte telomere length were all  obtained from a European population of 472,174  participants.  GWAS data for both sepsis and septic 28-day all cause death were obtained from the same European  population, including up to 486,484 participants | N/A | (sepsis to LTL),: β (SE) 0.008 (0.007), p=0.265) | N/A | Reverse, short telomere increases susceptibility to sepsis | No evidence supporting a genetically predicted causal relationship between 28 day sepsis death, Sepsis (critical care) and sepsis (28 day death in critical care). | N/A |
|  | Unspecified blood cells [1] | Change in TL [1] | Relative TL [1] | Q-PCR [1] | None [1] | Zribi, 2019* | Israel | Cohort | Unspecified blood cells | 40 | telomere length was grouped into three groups shorter, unchanged, longer. In shorter group 6/21 had sepsis, in unchanged group 1/8 had sepsis, in longer group 1/11 had sepsis | N/A | none | unclear | N/A | N/A |
| C. pneumoniae [1] | Leukocyte [1] | TL [1] | relative TL [1] | Q-PCR [1] | Linear regression (Beta coefficient) [1] | Aiello, 2017* | USA | cross-sectional | Leukocyte non-specific | 163 | Mean difference in TL between reference group (unexposed) and exposed group,−0·0393 {0·0285} p=0·17, t-test | −0·0412 (0·03) p=0.182 | age, race/ethnicity, sex, education level, pack-years of smoking, BMI (kg/m2), and diabetes history. | No | N/A | No interaction with sex found |
| Ebv [2] | Leukocyte [2] | TL and/ or change in TL [2] | Relative TL [2] | Q-PCR [2] | Linear regression (Beta coefficient) [2] | Yang, 2024* | Canada | Cross-sectional | Leukocyte | 376 | N/A | -0.04 (-0.25-0.18) | sex, 10 year age, HIV status, ethnicity, smoking, total no. non-HIV viruses, virus type | No | N/A | N/A |
|  |  |  |  |  |  | Dowd, 2017* | United Kingdom | Cohort and cross-sectional | Non-specific leukocyte | 400 | Not provided | follow-up LTL (ie cross-sectional): beta (SE) 0.009 (0.015) p=.52 , change in LTL (ie longitudinal): .0094477 (.0150655 SE) p=0.531 (95% CI -.0201736 to .0390691) | age, sex, smoking, employment grade, categorical body mass index, and baseline LTL and mutually adjusted for the other infections | No | Higher EBV IgG antibody levels among those seropositive was also associated with shorter LTL (β = −0.023 [0.009]; P = .02) | “ No significant sex-infection interactions were found” |
| Hhv-6 [1] | Leukocyte [1] | TL and change in TL [1] | relative TL [1] | Q-PCR [1] | Linear regression (Beta coefficient) [1] | Dowd, 2017* | United Kingdom | Cohort and cross-sectional | Non-specific leukocyte | 400 | Not provided | (note in non mutually adjusted model this was ‘significant’ ie p<0.05) follow-up LTL (ie cross-sectional): beta (SE) −0.027 (0.014) p=0.06 , change in LTL (ie longitudinal): -.022 (.015 SE) p=0.131 (95% CI -.0517075 to .0067464) | age, sex, smoking, employment grade, categorical body mass index, and baseline LTL and mutually adjusted for the other infections | Weak evidence | No significant associations were found for HHV-6 (β = −0.019 [0.013]; P = .16) IgG levels | “ No significant sex-infection interactions were found” |
| Hhv-8 [1] | Leukocyte [1] | TL and change in TL [1] | relative TL [1] | Q-PCR [1] | Linear regression (Beta coefficient) [1] | Yang, 2024* | Canada | Cross-sectional | Leukocyte | 376 | N/A | 0.19 (-0.003-0.39) | sex, 10 year age, HIV status, ethnicity, smoking, total no. non-HIV viruses, virus type | No | N/A | N/A |
| Hsv-2 [2] | Leukocyte [2] | TL [2] | Log relative TL [2] | Q-PCR [2] | Linear regression (Beta coefficient) [2] | Noppert, 2020* | USA | cross-sectional | Leukocyte non-specific | 1708 | Not provided | -0.0001 (-0.04, 0.04) p=0.99 | age, gender, race, education, BMI, cigarette smoking status, CRP, white blood cell count | No | N/A | No evidence for interaction with age or sex was found. |
|  |  |  |  |  |  | Yang, 2024* | Canada | Cross-sectional | Leukocyte | 376 | N/A | 0.07 (-0.12-0.27) | sex, 10 year age,HIV status, ethnicity, smoking, total no. non-HIV viruses, virus type | no | N/A | N/A |
| HTLV-1 [1] | PBMCs [1] | TL [1] | relative TL [1] | Q-PCR [1] | Unpaired t-test (No effect estimate) [1] | Usadi, 2016* | USA | Cross-sectional | PBMCs | 135 | 0.99 +- [0.18] vs 1.02 +- [0.16] (p = 0.322, comparing combined HTLV-1 & 2 to controls, Unpaired t-test) | N/A | matched on age, sex and race/ethnicity | No | No “significant” association of pro viral load and TL | N/A |
| HTLV-2 [1] | PBMCs [1] | TL [1] | relative TL [1] | Q-PCR [1] | Unpaired t-test (No effect estimate) [1] | Usadi, 2016* | USA | Cross-sectional | PBMCs | 135 | 0.99 +- [0.18] vs 1.03 +- [0.17] (p = 0.322 (p = 0.322, comparing combined HTLV-1 & 2 to controls, Unpaired t-test) | N/A | matched on age, sex and race/ethnicity | No, severity results using multivariable linear regression telomere length vs pro-viral load also confirmed this | No “significant” association of pro viral load and TL | N/A |
| Helminths [1] | Leukocyte [1] | TL [1] | Relative TL [1] | Q-PCR [1] | Linear regression (β coefficient) [1] | Macamo, 2024* | South Africa | Cross-sectional | Leukocyte | 100 | 1.21 ± [0.53] vs 0.83 ± [0.33] | -0.51 (-0.84 - -0.18)  Note: this was the fully-adjusted model, there was 3 other models with various levels adjustment | Age, Gender, Body Mass Index (BMI), HIV status, Employment, Income source/Occupation, Monthly income, Education, Intake of alcohol per day, Intake of drugs or marijuana, Water source, Toilet type, Present diseases aware of, Any illness in the past 30 days, Previous worm infection, Deworming medication in the past 6 months, Allergic reaction in the past 30 days, Chronic illness, Medication taken, Supplements taken, Antibiotics/Probiotics taken in the past 2 weeks, Tuberculosis (TB) infected, Antiretroviral therapy,  Iron status markers, Serum minerals and proteins, Inflammatory and nutritional markers, Full blood count parameters. | Yes | N/A | N/A |
| Rhinovirus [1] | Specific leukocytes [1] | TL [1] | Absolute TL [1] | FLOW-FISH [1] | Ordinary least squares regression ( Ordinary least squares effect) [1] | Andreu-Sanchez, 2024* | Netherlands | Cross-sectional | Lymphocytes, Granulocytes, Naive T-cells, Memory T-cells, B-cells, NK-cells | 1,243 | N/A | Ordinary least squares effect (standard error, p-value)  Lymphocytes: 0.019 (0.0042, p = 6.3 × 10⁻⁶)  Granulocytes: 0.012 (0.0045, p = 0.0088) Naive T-cells:, 0.020 (0.0045, p = 1.5 × 10⁻⁵) Memory T-cells:0.020 (0.0045, p = 1.5 × 10⁻⁵) , B-cells: 0.013 (0.0041, p = 0.0012) , NK-cells: 0.017 (0.00465, p = 2.66 × 10⁻⁴) | Unclear | Yes | N/A | N/A |
| HCV-HIV coinfection [1] | PBMCs [1] | Change in TL [1] | median telomere length of T cell subsets relative to telomere length of calf thymocytes [1] | FLOW-FISH [1] | Unclear (No effect estimate) [1] | Grady, 2013* | Netherlanda | Cohort | CD8+ and CD4+ T-cells | N=74,  4 groups including 1. healthy individuals (n = 22) , 2. Drug-users who were Multiple Exposed (to HCV) Uninfected (MEU) (n = 8);  3. chronic HCV (cHCV) monoinfected drug-users (n =21); 4. cHCV/HIV coinfected drug-users (n = 23). | Cd8+ T-cells  Cd8+ T-cells decreased from a median RTL of 0.36 (0.24–0.33 IQR) to 0.28 ( 0.24– 0.33) in MEU, 0.36 (IQR 0.32–0.41) to 0.30 (IQR 0.26–0.34) in cHCV monoinfected and 0.30 (0.27–0.30) to 0.23 (0.21–0.28) in cHCV/HIV coinfected drug-users.   Telomere length of Cd8+ T-cells in HIV/cHCV infected individuals at time point 2 was shorter compared to those with cHCV infection, MEU or healthy controls, p < 0.01  Cd4+ T-cells  “Telomere length of Cd4+ T-cells in HIV/cHCV coinfection was significantly (RTL 0.26, IQR 0.23–0.31) reduced as compared to healthy donors (RTL 0.31, IQR 0.27–0.34), p < 0.05” | N/A | None | yes | N/A | N/A |
| Combined Burden of HSV-1,HSV-2, CMV, H.Pylori, HBV [1] | Leukocyte [1] | TL [1] | Log relative TL [1] | Q-PCR [1] | Linear regression (Beta coefficient) [1] | Noppert, 2020* | USA | cross-sectional | Leukocyte non-specific | 1708 | Not provided | 1 pathogen -0.03 (-0.07, 0.02), 2-3 pathogen -0.03 (-0.07, 0.02), 4+ pathogens 0 (-0.04, 0.05  Note: there was also ‘composite’ score variable the results of which are not included here as this review focused on numerical burden. | age, gender, race, education, BMI, and cigarette smoking status | No | See adjusted results column | No statistical test for interaction was performed , but found that men with 1 to 3 pathogen burden had significantly reduced leukocyte telomere length in men compared to uninfected male controls. Not found in women. |
| Combined burden of C. pneumoniae, HSV-1, CMV, H.Pylori [1] | Leukocyte [1] | TL [1] | relative TL [1] | Q-PCR [1] | Linear regression (Beta coefficient) [1] | Aiello, 2017* | USA | cross-sectional | Leukocyte non-specific | 163 | Low burden (0-2 pathogens) : 0·917 {0·159} vs high burden (2-4 pathogens) : 0·900 {0·166}, p=0.6 T test | −0·0367 (0·0354) p=301 (high burden vs low) | age, race/ethnicity, sex, education level, pack-years of smoking, BMI (kg/m2), and diabetes history. | No | Increased total pathogen burden level was significantly associated with shorter telomere length among females (β=−0·1057 (S.E. = 0·05), P= 0·033) linear regression fully adjusted model | N/A |
| Combined burden of CMV, HSV-1, HSV-6, EBV [1] | Leukocyte [1] | TL and change in TL [1] | relative TL [1] | Q-PCR [1] | Linear regression (Beta coefficient) [1] | Dowd, 2017* | United Kingdom | Cohort and cross-sectional | Non-specific leukocyte | 400 | Not provided | Cross-sectional  1-2 pathogens did not show associations versus 0 pathogens at baseline (cross-sectional analysis) or longitudinal analysis.  However 3 vs 0 pathogens at baseline gave a beta coefficient of 0.069 {0.029} p= 0.02.  4 v 0 pathogens at Baseline gave β=−0.139 {0.037} p<0.001  Longitudinal  beta coefficients for longitudinal results are as follows for 3 vs 0 pathogens : -0.0608 (-0.1198 to -0.00178) p= 0.044  and for 4 vs 0 pathogens:  -0.1255 (-0.2000 to -0.0509) p=0.001 | age, sex, smoking, employment grade, and baseline LTL | 3 and 4 pathogens yes, 1-2 no | See results column | “ No significant sex-infection interactions were found” |
| Helminths + HIV coinfection [1] | Leukocyte [1] | TL [1] | Relative TL [1] | Q-PCR [1] | Linear regression (β coefficient) [1] | Macamo, 2024* | South Africa | Cross-sectional | Leukocyte | 100 | 1.21 ± [0.53] vs 0.93 ± [0.41] | -0.35 (-1.05 - 0.35)  Note: this was the fully-adjusted model, there was 3 other models with various levels adjustment some of which did show evidence of association | Age, Gender, Body Mass Index (BMI), HIV status, Employment, Income source/Occupation, Monthly income, Education, Intake of alcohol per day, Intake of drugs or marijuana, Water source, Toilet type, Present diseases aware of, Any illness in the past 30 days, Previous worm infection, Deworming medication in the past 6 months, Allergic reaction in the past 30 days, Chronic illness, Medication taken, Supplements taken, Antibiotics/Probiotics taken in the past 2 weeks, Tuberculosis (TB) infected, Antiretroviral therapy,  Iron status markers, Serum minerals and proteins, Inflammatory and nutritional markers, Full blood count parameters. | No/ mixed | N/A | N/A |
| Combined burden of CMV, EBV, HHV-8, HSV-1, HSV-2, HCV [1] | Leukocyte [1] | TL [1] | Relative TL [1] | Q-PCR [1] | Linear regression (β coefficient) [1] | Yang, 2024* | Canada | Cross-sectional | Leukocyte | 376 | N/A | Total # of non-HIV viruses (vs. 0-2)  3-4: -0.26 (-0.47- -0.04)  5-6: 0.08 (-0.41-0.57) | sex, 10 year age,HIV status, ethnicity, smoking, total no. non-HIV viruses, virus type | mixed | N/A | N/A |

Footnotes

1. This study self-identifies as a cross-sectional study but also presents results of ‘percentage change’ it is unclear how this longitudinal measure is incorporated into the study design.
2. These results extracted from figure 1 (Breen, 2022)
3. Cross-sectional analysis within a randomised controlled trial
4. Study states it measured ‘relative’ telomere length, it is unclear why units are in kilobase pairs.

*- ‘Unique’ E-O relationships are marked with asterix. For each infection, one E-O relationship from each overlapping sample was classed as ‘unique’. To determine the unique E-O relationship, priority was given to those with longitudinal designs, followed by studies with the greatest sample size and to the most recent study.

Superscript letters (a–k) indicate E-O relationships within the same population for a specific pathogen or infection, those with the same letter have the same/overlapping samples.

**Table abbreviations:**

TL = Telomere length, Q-PCR = Quantitative Polymerase Chain Reaction, TRF = Terminal Restriction Fragment, PBMCs = Peripheral Blood Mononuclear Cells, SNP = Single Nucleotide Polymorphism, MR = Mendelian Randomization, ART = Antiretroviral Therapy, CMV = Cytomegalovirus, HCV = Hepatitis C Virus, HBV = Hepatitis B Virus, HPV = Human Papillomavirus, HTLV = Human T-Lymphotropic Virus, NHANES = National Health and Nutrition Examination Survey, ELISA = Enzyme-Linked Immunosorbent Assay, FISH = Fluorescence In Situ Hybridization, T/S Ratio = Telomere-to-Single Copy Gene Ratio, DNAmTL = DNA methylation-based telomere length
