# Supplementary material for: Infection and telomere length: A systematic review
Source: PLoS One. 2025 Sep 23;20(9):e0333107. doi: 10.1371/journal.pone.0333107 (PMC12456831; doi:10.1371/journal.pone.0333107)
Supplement: Attachment — Submitted filename: Rebuttal letter.docx [file pone.0333107.s009.docx]

**Response to Reviewers**
Manuscript ID: PONE-D-25-38272
Title: *Infection and telomere length: a systematic review*

Dear Dr. Saretzki,

We would like to thank you and the reviewer for your thoughtful and constructive feedback on our manuscript. We appreciate the opportunity to revise our work and have carefully addressed all points raised by the academic editor, the reviewer, and the journal’s requirements. Below we provide a detailed, point-by-point response.

**Reviewer #1 Comments**

**Major Point 1:**
*It has been shown that individuals bearing shorter TL are more susceptible to infection of different viruses or associated with more adverse outcomes than those with longer TL (JAMA, 2013;309:699; Cell Mol Life Sci, 2022;79:110; EBioMedicine. 2021;70:103485). Thus, the relationship between infection and TL may be reciprocal, like “the chicken or egg causality dilemma”. The authors are suggested to discuss this issue.*

**Response:**
We thank the reviewer for their valid point, we have addressed this issue by adding the following to the discussion section:
‘*Most included studies were cross-sectional , which limits the ability to establish causality between infection and TL. Longitudinal studies are essential for determining whether infections lead to telomere shortening or if individuals with shorter telomeres are more susceptible to certain infections. Previous studies have suggested the latter, with shorter TL associated with greater susceptibility to viral infection and worse clinical outcome (97,98), highlighting the possibility of a reciprocal relationship between infection and TL akin to the “chicken or egg” dilemma.*’

**Minor Point 1:**
*The abbreviation “TA” usually indicates telomerase activity, which might make readers confused here (at least for reviewer). In addition, TA is not in the abbreviation list.*

**Response:**
We have now removed the abbreviation “TA” and instead used the unabbreviated ‘telomere attrition’ throughout the manuscript.

**Journal Requirements**

**1. Manuscript style requirements (formatting, file naming).**
**Response:**
We have now amended the naming and formatting of files, figures and tables according to the PLOSONE criteria.

**2. Financial disclosure – role of funders.**
**Response:**
The funders had no role in study design, data collection and analysis, decision to publish, or preparation of the manuscript.

**3. Separate captions for each figure.**
**Response:**
We now have separate captions for each figure in-line with the PLOSONE criteria.

**4. Captions for** **Supporting Information files and consistent in-text citations.**
**Response:**
We now have captions for supporting information and have consistent in-text citations.

**5. Fully anonymised data set, removal of personal information.**
**Response:**
With regard to the request to remove personal information and provide an anonymised dataset, we would like to clarify that our manuscript is a systematic review and does not involve primary data collection or human participants. As such, no participant-level data are included, and the requirement to anonymise datasets is not applicable in this case.

**6. Reviewer-suggested references – evaluation and citation if relevant.**
**Response:**

We have reviewed the cited publications. Cohen et al. (JAMA, 2013) and Wang et al. (EBioMedicine, 2021) are directly relevant, as they provide evidence that shorter telomere length is associated with increased susceptibility to viral infection and adverse outcomes. These have now been cited in the Limitations section, where we discuss the possibility of a reciprocal relationship between infection and telomere length.

**7. Reference list accuracy, completeness, and handling of retracted papers.**
**Response:**
We have checked the reference list and the status (retracted or not) of cited papers, we confirm that there are no retracted papers and that the reference list is complete and accurate.

**Additional Editor Comments**

*Please address the comments of the reviewer carefully.*

**Response:**
We believe we have carefully addressed all reviewer comments, making changes to the manuscript where needed.

We believe these revisions have strengthened the manuscript, and we are grateful to the editor and reviewer for their guidance. We look forward to your consideration of our revised submission.

Sincerely,
Louis Tunnicliffe
on behalf of all authors
